# Activated HLA-DR^+^CD38^+^ Effector Th1/17 Cells Distinguish Crohn’s Disease-associated Perianal Fistulas from Cryptoglandular Fistulas

**DOI:** 10.1093/ibd/izae103

**Published:** 2024-05-22

**Authors:** Laura F Ouboter, Ciska Lindelauf, Qinyue Jiang, Mette Schreurs, Tamim R Abdelaal, Sietse J Luk, Marieke C Barnhoorn, Willem E Hueting, Ingrid J Han-Geurts, Koen C M J Peeters, Fabian A Holman, Frits Koning, Andrea E van der Meulen-de Jong, Maria Fernanda Pascutti

**Affiliations:** Department of Gastroenterology and Hepatology, Leiden University Medical Center, Leiden, the Netherlands; Department of Immunology, Leiden University Medical Center, Leiden, the Netherlands; Department of Immunology, Leiden University Medical Center, Leiden, the Netherlands; Department of Immunology, Leiden University Medical Center, Leiden, the Netherlands; Department of Immunology, Leiden University Medical Center, Leiden, the Netherlands; Department of Radiology, Leiden University Medical Center, Leiden, the Netherlands; Bioinformatics Lab, Delft University of Technology, Delft, the Netherlands; Systems and Biomedical Engineering Department, Faculty of Engineering Cairo University, Giza, Egypt; Department of Hematology, Leiden University Medical Center, Leiden, the Netherlands; Department of Gastroenterology and Hepatology, Leiden University Medical Center, Leiden, the Netherlands; Department of Surgery, Alrijne hospital, Leiderdorp, the Netherlands; Department of Surgery, Proctos Clinic, Bilthoven, the Netherlands; Department of Surgery, Leiden University Medical Center, Leiden, the Netherlands; Department of Surgery, Leiden University Medical Center, Leiden, the Netherlands; Department of Immunology, Leiden University Medical Center, Leiden, the Netherlands; Department of Gastroenterology and Hepatology, Leiden University Medical Center, Leiden, the Netherlands; Department of Immunology, Leiden University Medical Center, Leiden, the Netherlands

**Keywords:** high-dimensional analyses, perianal fistulas, Crohn’s disease

## Abstract

**Background:**

Perianal fistulas are a debilitating complication of Crohn’s disease (CD). Due to unknown reasons, CD-associated fistulas are in general more difficult to treat than cryptoglandular fistulas (non-CD-associated). Understanding the immune cell landscape is a first step towards the development of more effective therapies for CD-associated fistulas. In this work, we characterized the composition and spatial localization of disease-associated immune cells in both types of perianal fistulas by high-dimensional analyses.

**Methods:**

We applied single-cell mass cytometry (scMC), spectral flow cytometry (SFC), and imaging mass cytometry (IMC) to profile the immune compartment in CD-associated perianal fistulas and cryptoglandular fistulas. An exploratory cohort (CD fistula, n = 10; non-CD fistula, n = 5) was analyzed by scMC to unravel disease-associated immune cell types. SFC was performed on a second fistula cohort (CD, *n* = 10; non-CD, *n* = 11) to comprehensively phenotype disease-associated T helper (Th) cells. IMC was used on a third cohort (CD, *n* = 5) to investigate the spatial distribution/interaction of relevant immune cell subsets.

**Results:**

Our analyses revealed that activated HLA-DR^+^CD38^+^ effector CD4^+^ T cells with a Th1/17 phenotype were significantly enriched in CD-associated compared with cryptoglandular fistulas. These cells, displaying features of proliferation, regulation, and differentiation, were also present in blood, and colocalized with other CD4^+^ T cells, CCR6^+^ B cells, and macrophages in the fistula tracts.

**Conclusions:**

Overall, proliferating activated HLA-DR^+^CD38^+^ effector Th1/17 cells distinguish CD-associated from cryptoglandular perianal fistulas and are a promising biomarker in blood to discriminate between these 2 fistula types. Targeting HLA-DR and CD38-expressing CD4^+^ T cells may offer a potential new therapeutic strategy for CD-related fistulas.

**Key Messages**

**What is already known?**

T helper CD4^+^ T cells are crucial drivers in the pathogenesis of Crohn’s disease, but their role in perianal fistulas is understudied.

**What is new here?**

We, for the first time, performed high-dimensional single-cell and spatial interaction analyses of CD-associated and cryptoglandular perianal fistulas, revealing activated HLA-DR^+^CD38^+^ effector Th1/17 cells as distinctly increased in CD-associated compared with cryptoglandular fistulas, closely interacting with other (activated) effector CD4^+^ T cells and B cells, pointing to the potential function of this cell subset in the inflamed fistula tracts of CD patients.

**How can this study help patient care?**

Monitoring HLA-DR^+^CD38^+^ CD4^+^ T cells holds significant clinical promise for guiding treatment strategies due to the distinct treatment approaches for CD-associated and cryptoglandular perianal fistulas and the potential for fistulas to serve as initial disease manifestations.

## Introduction

Crohn’s disease (CD), one of the subtypes of inflammatory bowel diseases (IBDs), is a chronic, immune-mediated disease. In patients with IBD, mucosal integrity of the intestine is often compromised, leading to translocation of luminal antigens and microbiota across the epithelial barrier.^1^ Chronic inflammation is believed to arise from a dysregulated mucosal immune response to these microbial antigens.^2^ Perianal fistulas, pathological connections between the intestinal lumen and the perianal skin, are a debilitating complication of CD. Up to 26% of patients with CD suffer from perianal fistulizing disease within 20 years of initial diagnosis.^3-5^

More than 90% of all perianal fistulas are unrelated to IBD and may arise from infected anal glands and the onset of perianal abscesses and are hence called cryptoglandular.^6-8^ Crohn’s disease–associated and cryptoglandular perianal fistulas look similar macroscopically but differ considerably in complexity, treatment strategies, and healing rates.^9^ Distinction between these 2 types of fistulas is mainly based on the presence or absence of (previous) luminal inflammation. However, in 30% of the patients with CD, development of a fistula is the first manifestation and precedes luminal disease by years.^10^ In these cases, identifying CD-associated from cryptoglandular perianal fistulas might be challenging. In general, cryptoglandular fistulas are managed surgically, whereas CD-associated fistulas require combined surgical and medical approaches and still have a poor success rate.^11,12^ Most patients with CD-associated fistulas receive medical treatment with antitumor necrosis factor (TNF)-α, but their effectiveness is limited, with over 60% of patients that relapse after 1 year of maintenance therapy.^13,14^ Thus, it is crucial to differentiate between the 2 types of fistulas in order to initiate the appropriate treatment trajectory, and concurrently, it is imperative to identify novel targets for therapy-refractory patients.

The pathogenesis of CD-associated fistulas is still elusive but likely involves host, microbial, and environmental factors. It has been speculated that luminal bacterial stimuli trigger an aberrant immune response that leads to persistent mucosal inflammation in a genetically predisposed individual. Due to an aberrant immune response triggered by luminal bacterial antigens, cytokines, including TNF-α, transforming growth factor (TGF)-β, and interleukin (IL)-13, induce transcription factors that promote epithelial-mesenchymal transition (EMT) and cell invasion leading to fistula formation.^15^ An inflammatory process also appears to play a crucial role in cryptoglandular fistulas next to the presence of EMT, but this is still understudied.^16,17^

The immune cell composition of the immune infiltrates in both fistula types is still poorly characterized. Understanding the differences in the immune cell subsets present in the fistula tract between CD-associated and cryptoglandular fistulas could help us understand the underlying biological mechanisms contributing to fistula persistence.

High-dimensional single-cell mass cytometry (scMC) allows the simultaneous identification and characterization of immune cell populations across multiple lineages, providing the opportunity to explore the mucosal immune system at great depth.^18^ With this technique, we performed a comprehensive characterization of the immune cell landscape in perianal fistulas of CD-diagnosed patients and patients not diagnosed with CD. Peripheral blood mononuclear cell (PBMC) samples were also collected from these patients to explore if particular immune cell populations residing in the fistula were also present in blood. Additionally, a second cohort was obtained to investigate, by spectral flow cytometry (SFC), the proliferation, regulation, and differentiation state of HLA-DR^+^CD38^+^ CD4^+^ effector memory T (T_EM_) cells and CD8^+^ T_EM_ cells associated with CD fistulas. Lastly, a third cohort was used for the analysis of cellular distribution patterns by imaging mass cytometry (IMC). The data generated in this work were used to further dissect the inflammatory process in fistulas and its drivers with the ultimate goal to provide insights for therapeutic approaches.

## Materials and Methods

### Overview of the Study

Firstly, we employed scMC to obtain a comprehensive immune cell landscape in perianal fistulas of patients with and without CD. In this cohort (cohort 1), we included 21 patients (CD, *n *= 14; non-CD, *n* = 7) undergoing surgical intervention at the Leiden University Medical Center (LUMC, Leiden, the Netherlands), the Proctos clinic (Bilthoven, the Netherlands), and the Alrijne hospital (Leiderdorp, the Netherlands), for their perianal fistula between July 2019 and June 2021. Curettage material of perianal fistulas, rectum biopsies, and peripheral blood mononuclear cell (PBMC) samples were collected and freshly processed. Some samples were excluded for further analysis due to a low number of total CD45^+^ cells (<1000; CD-fistula, *n* = 3; non-CD-fistula, *n* = 2; CD-rectum, *n* = 1; non-CD-rectum, *n* = 2), bad quality of the sample (CD-fistula, *n* = 1; CD-rectum, *n* = 1), or were not obtained because no patient consent for rectum sampling was given (non-CD-rectum, *n* = 1). Ultimately, we analyzed curettage material of 15 fistulas (CD, *n* = 10; non-CD = 5), 16 rectum biopsies (CD, *n* = 12; non-CD, *n* = 4), and 21 PBMC (CD, *n* = 14; non-CD, *n* = 7) samples. Secondly, based on our findings in the scMC cohort, SFC was used to perform deep T cell phenotyping including the identification of the distinct T helper subsets. For this purpose, curettage material of perianal fistulas from a new cohort of CD patients (*n* = 10, 3 of whom were also included in cohort 1), and non-CD patients (*n* = 11) was collected during surgery at the LUMC, the Proctos Clinic and the Alrijne hospital from December 2021 to October 2022. After collection, material of this second cohort (cohort 2) was cryopreserved in freezing medium (20%FCS/60%RPMI/20%DMSO) for staining at a later time point.

Thirdly, we examined the interaction between immune cells using formalin-fixed paraffin-embedded (FFPE) samples (*n* = 8) obtained from fistula tracts of a third cohort (cohort 3) of a total of 5 CD patients. We obtained 3 samples from one patient and 2 samples from another patient, as we had 3 and 2 tissue blocks, respectively, from the same fistula tract (same time point). For each sample, 2 consecutive slides were prepared and stained; one with Haematoxylin and Eosin (H&E) to determine immune cells around the tract and to select regions of interest (ROI) and another with a 40-marker antibody panel for IMC.

**Figure 1.**
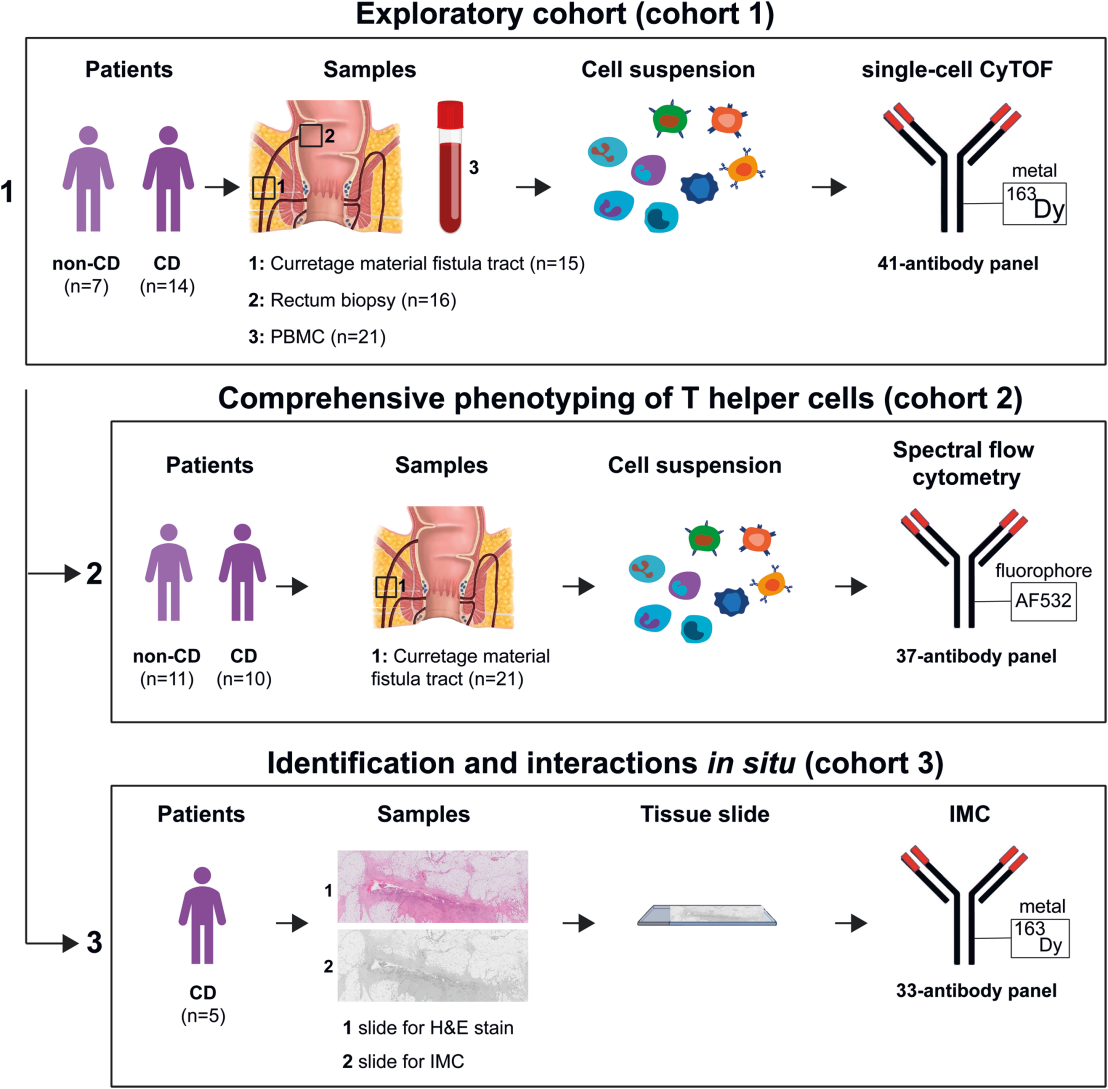
Study design. Three different techniques were applied to samples from fistula-bearing patients with and without CD to unravel the immune cell landscape in perianal fistulas (single-cell mass cytometry; cohort 1; top row), the T (helper) phenotype of activated T cells associated with CD fistulas (spectral flow cytometry; cohort 2; middle row), and interaction of immune cells in situ (imaging mass cytometry; cohort 3; bottom row). Abbreviations: CD, Crohn’s disease; PBMC, peripheral blood mononuclear cell; Dy, dysprosium; H&E, hematoxylin and eosin; IMC, imaging mass cytometry; AF, alexa fluor.

An overview of the study design and the patients’ cohorts is displayed in Figure 1 and Supplementary Table 1, respectively.

### Human Samples

Patients eligible for inclusion were 18 years or older with an indication for incision and drainage of their perianal fistula, regardless of the type of perianal fistula (according to Parks’ classification^19^) and number of fistulas or side-branches. Patients with rectovaginal fistulas or with a fistula after radiotherapy were excluded. The material was collected during surgery and further processed at the Department of Immunology of the LUMC. The medical data of the patients were retrieved from the electronic medical record. For the exploratory scMC cohort (cohort 1), fistula material was curetted via the external opening of the tract. The material within the tract was scraped via the external orifice of the fistula tract, which was dilated for this purpose. Two rectum biopsies were taken around the internal opening, and 16 mL of blood was drawn. For SFC (cohort 2), only fistula curettage material was used. Formalin-fixed paraffin-embedded samples (cohort 3) of fistula tracts were obtained from the pathology archive. The Medical Ethical Committee of the Leiden University Medical Center (LUMC) approved this entire study (protocol P18.069), and patients provided written informed consent. All specimens were anonymized and handled in accordance with the local ethical guidelines for the LUMC and in accordance with the Declaration of Helsinki.

## Experimental Methods

### Sample Processing

In cohort 1, leukocytes were isolated from fresh rectal biopsies and fistula scrapings and stained with a 41-antibody panel (Supplementary Table 2) for scMC. For SFC (cohort 2), the cryopreserved isolated cells were stained with a 37-antibody panel (Supplementary Table 3). A detailed description of the sample processing is provided in the Supplementary Methods.

In cohort 3, IMC was applied to FFPE fistula specimens. These samples were obtained during fistulectomy. Formalin-fixed paraffin-embedded samples from fistula tracts were cut with a microtome (Leica RM225, Bannockburn, IL, USA) into 4-μm sections and were subsequently deparaffinized. The location of the fistula tract was determined together with the pathologist. Tissue sections were prepared for immunodetection by IMC and stained with a 33-antibody panel (Supplementary Table 4) followed by laser ablation coupled to mass spectrometry to generate high-dimensional images as previously described.^20^

### Antibodies, Antibody Staining, and Data Acquisition

Antibodies, manufacturers, and concentrations are listed in Supplementary Tables 2, 3, and 4 for scMC, SFC, and IMC, respectively. For scMC and IMC, preconjugated metal-labeled antibodies were either purchased from Fluidigm (San Francisco, California, USA) or conjugated in-house using 100 mg of carrier-free formulations of purified antibody combined with the MaxPar X8 antibody labeling kit (Fluidigm Sciences) according to the manufacturer’s instructions. Following conjugation, antibodies were diluted to 200 mL in Candor phosphate-buffered saline (PBS) antibody stabilization buffer (Candor BioscienceGmbH, Wangen im Allgäu, Germany) and stored at 4 °C. Self-conjugated antibodies were validated using OneComp beads (Invitrogen, Waltham, Massachusetts, USA) and on PBMCs. Further details on the antibody staining and data acquisition are described in the Supplementary Methods.

### Data Analysis

#### Single-cell CyTOF

**Figure 2.**
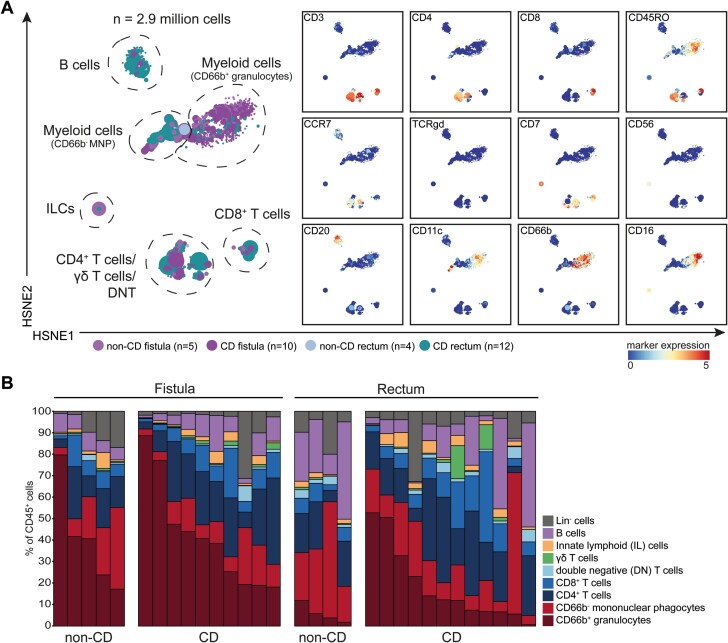
Similar composition of the immune compartment in fistula tracts from patients with Crohn’s disease and patients without Crohn’s disease. A, HSNE embedding showing 2.3 × 10^3^ landmarks representing immune cells (2.9 × 10^6^) isolated from fistula scraping samples (*n* = 15), rectum biopsies (*n* = 16) from patients with Crohn’s disease (*n* = 14) and patients without Crohn’s disease (*n* = 7). Colors represent the different samples (left) and marker expression (right). B, Composition of the major immune lineages in fistula scraping samples (*n* = 15) and rectum biopsies (*n* = 16), represented in vertical bars. The size of the colored segments represents the proportion of the cells as a percentage of total CD45^+^ cells in the samples.

First, for data cleaning, all samples were pregated, using the FlowJo v10 Software, on single, live CD45^+^ cells, removing duplicates, beads, dead cells, and debris. A reference PBMC sample was included in each experiment to account for technical variation. All reference PBMC samples were obtained from blood from the same healthy individual from whom cells were isolated and aliquoted. ComBat was applied to align the PBMC reference samples and corresponding patient samples to correct for batch effects.^21^ The markers CD66b, CD15, Nkp44, c-kit, CD40, CD80, and PD-L1 were not present in the reference PBMCs at sufficient levels to scale and were thus not normalized. For further analysis, these antibodies were not used for clustering but were shown for marker expression overlays and interpretation of data; CD45^+^ cells were sample-tagged, hyperbolic ArcSinh transformed with a cofactor of 5, and subjected to dimensionality reduction analysis in Cytosplore.^22^ Major immune lineages (Figure 2A) were identified at the overview level of a 5-level hierarchical stochastic neighbor embedding (HSNE) analysis on CD45^+^ data from fistula samples and rectal biopsy samples with default perplexity and iterations (30 and 1000, respectively).^23,24^ The CD4^+^ T cells, CD8^+^ T cells, innate lymphoid cells (ILCs), and B cells were analyzed separately in a data-driven manner up to a maximum number of 0.5 × 10^6^ landmarks using *t*-distributed stochastic neighbor embedding (tSNE).^24^ One rectum sample within the CD4^+^ T cell analysis had a 10-times higher number of cells than the other samples; for that reason, we downsampled to the second-highest cell count. The CD66b^+^ granulocytes and CD66b^-^ myeloid cells were identified within the myeloid cell compartment at the overview level of a 3-level HSNE analysis. Additionally, CD66b^-^ myeloid cells were further analyzed using tSNE. Clustering of the data was performed by Gaussian mean shift (GMS) clustering in Cytosplore, and an algorithm was run that merged clusters showing high similarity in ArcSinh5-transformed median expression of all markers (<1). Quantification of frequencies of clusters in each sample was performed in GraphPad Prism v9. The R-package “corrplot”^25^ was used to calculate the correlation networks of cell frequencies of the identified cell subsets.

#### Spectral flow cytometry

First, similar to what was described for scMC data, SFC data from all samples were cleaned, using the FlowJo v10 software, by gating on single, live CD45^+^ cells. A time gate was used to remove events acquired during unstable windows of acquisition. Conventional T cells were gated as CD19^-^ CD7^+/-^ CD56^-^ CD3^+^ TCR_γδ_^-^ Va7.2^-^ cells and subgated as CD8_α_^+^ CD8_β_^+^ (conventional CD8_αβ_^+^ T cells) or CD8_α_^-^ CD8_β_^-^ FoxP3^-^ Helios^-^ CD4^+^ (conventional CD4^+^ T cells). Subsets of conventional CD8_αβ_^+^ and CD4^+^ T cells were gated based on CD45RA, CD45RO, and CCR7 expression. The full gating strategy is depicted in Supplementary Figure 6A-C.

#### Imaging mass cytometry

An imaging processing pipeline was used to generate cell masks to convert raw IMC images into single-cell data.^26^ This involved the creation of cell segmentation masks using semiautomated background identification with Ilastik^27^ and CellProfiler,^28^ after which the resulting masks were processed by ImaCytE^29^ for the generation of FCS files to obtain single-cell data. The t-SNE in Cytosplore was used to identify immune cell clusters. Cell clusters were merged based on overlapping markers and exported as separate FCS files. The resulting subsets were imported back into ImaCytE for visualization of subsets in the segmentation masks, and localization was compared with original MCD images to validate the obtained clusters. In ImaCytE, an overview of phenotypes in the microenvironment was obtained. Next to that, ImaCytE allowed exploration of the spatial interaction of phenotypes and identified which of the interactions were significant.

### Statistical Analyses

Data were presented as individual points with the median and interquartile range (IQR). Group comparisons were performed using the Mann-Whitney *U* (MWU) test (2-tailed; Graphpad prism v9). The MWU test–generated *P* values of the 124 immune cell clusters identified with the scMC analysis were subsequently adjusted for multiple hypothesis testing (FDR < 5%). To determine correlations between immune cell populations and to compare different tissues with each other, a Spearman’s rank correlation analysis was applied.

## Results

### Patient Characteristics

Baseline characteristics of all patients in cohort 1 and 2 are summarized in Supplementary Tables 5 and 6. In cohort 1, 85.7% of CD patients (*n* = 14) were females and were the median age 41 years. Of the non-CD patients (*n* = 7), 42.9% were females and were the median age 52 years. In cohort 2, 40% of CD patients (*n* = 10) were females and were the median age 41.5 years, and of the non-CD patients (*n* = 11), 27.3% were females and were the median age 44 years.

The median time since diagnosis of CD was 17.5 (cohort 1) and 12.0 (cohort 2) years, and the median time since diagnosis of the perianal fistula was 9.5 (cohort 1) and 6.0 (cohort 2) years. Biologics used for concomitant use included infliximab/adalimumab (cohort 1, *n* = 4; cohort 2, *n* = 5), ustekinumab (cohort 1, *n* = 4; cohort 2, *n* = 2), vedolizumab (cohort 1, *n* = 3; cohort 2, *n* = 0), and both ustekinumab and vedolizumab (cohort 1, *n* = 1; cohort 2, *n* = 0).

Of the non-CD patients, no one used biologics. The median time since diagnosis of a fistula was 1.0 year in both cohorts 1 and 2. In 3 of 7 patients in cohort 1 and in 1 of 11 patients in cohort 2, a colonoscopy was performed to exclude CD diagnosis, while the remaining had no colonoscopy as they had a negative anamnesis focused on CD-typical symptoms, extraintestinal manifestations (EIMs) of CD, presence of immune-mediated diseases (IMIDs), and patients’ family history according to our recently published flowchart.^15^ The feces calprotectin (FCP) value was not known.

No clinical information was accessible for the 5 patients in cohort 3. However, it was established that these patients had been diagnosed with CD and had undergone surgery for perianal disease.

### Similar Presence of the Major Immune Cell Subsets in Fistula Tracts From Patients With and Without CD

The heterogeneity of the innate and adaptive immune system in perianal fistulas and rectum biopsies from 14 patients (exploratory cohort; cohort 1) with CD and 7 patients without CD was deciphered by measuring 41 immune cell markers by scMC. Hierarchical stochastic neighbor embedding in Cytosplore was applied to the entire data set encompassing 2.9 million CD45^+^ cells from the fistula tract (1.6 million cells) and rectum biopsies (1.3 million cells) to obtain a global overview of the major immune lineages. Based on lineage marker expression, we could distinguish CD4^+^ and CD8^+^ T cells, TCRγδ T cells, double-negative T (DNT) cells, B cells, innate-lymphoid cells (ILCs), granulocytes (CD66b^+^), and mononuclear phagocytes (CD66b^-^, MNPs; Figure 2A). Quantification of cell frequencies revealed that the CD4^+^ T cells and the myeloid cells were the most dominant cell types in the fistula tract (Figure 2B and Supplementary Figure 2), the latter to a large part composed of granulocytes. At this level of analysis, however, we did not observe significant differences between CD and non-CD patients in both fistula tracts and rectum biopsies with regard to the presence of the major immune lineages. Next, we individually analyzed the HSNE-defined major immune lineages at the single-cell level with t-SNE.

### HLA-DR^+^CD38^+^ Effector CD4^+^ T and CD8^+^ T Cells Are Significantly Increased in CD-associated Fistulas Compared With Cryptoglandular Fistulas

We next used t-SNE to further explore the cellular heterogeneity within each major immune cell lineage. The embedding of the CD4^+^ T cell, CD8^+^ T cell, MNP, and ILC/NK cell compartment is shown in Supplementary Figure 3A-D. In total, this yielded 124 phenotypically distinct immune cell subsets in the fistula tract samples and rectum biopsies.

**Figure 3.**
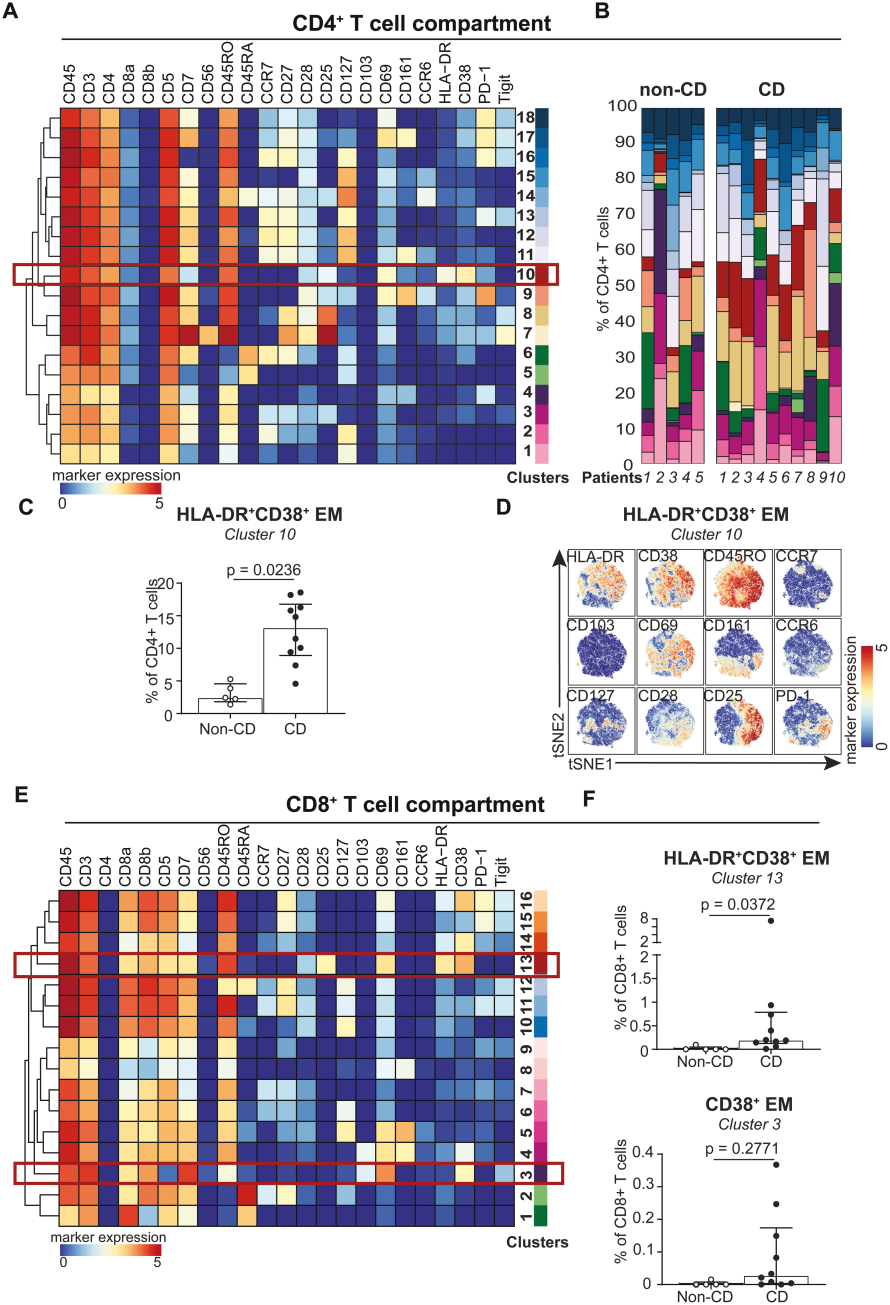
HLA-DR^+^CD38^+^ effector CD4^+^ T and CD8^+^ T cells are significantly increased in Crohn’s disease-associated perianal fistulas compared with cryptoglandular fistulas. A, Heatmap of CD4^+^ T cell subsets identified through differential expression of the phenotypic markers indicated. The color indicates the median ArcSinh5-transformed marker expression values (blue-to-red scale) of the clusters identified in Supplementary Figure 3A (cluster partitions). B, Composition of the CD4^+^ T cell compartment in cryptoglandular fistulas (*n* = 5) and CD-associated fistulas (*n* = 10) represented in vertical bars where the size of the colored segments represents the proportion of cells as a percentage of total CD4^+^ in the sample. Colors for each cluster as in A. C, Frequencies of the one significant subset out of a total of 18 CD4^+^ T cell subsets among cryptoglandular fistulas (*n* = 5) and CD-associated fistulas (*n* = 10) as percentage of total CD4^+^ T cells. Cluster numbers correspond to the ones in (A). Bars indicate median with interquartile range. Each dot represents an individual sample. Mann-Whitney *U* test with correction for multiple testing with a False Discovery Rate of 1% was performed. D, A t-SNE embedding showing the HLA-DR^+^CD38^+^ CD4^+^ T cells (61 000 cells) derived from rectum biopsies and fistula scrapings from CD patients and non-CD patients. E, Heatmap of CD8^+^ T cell subsets identified through differential expression of the phenotypic markers indicated. The color indicates the median ArcSinh5-transformed marker expression values (blue-to-red scale) of the clusters identified in Supplementary Figure 3B (cluster partitions). F, Frequencies of the one significant subset (top) and the subset that showed a trend out of a total of 16 CD8^+^ T cell subsets among cryptoglandular fistulas (*n* = 5) and CD-associated fistulas (*n* = 10) as percentage of total CD4^+^ T cells. Cluster numbers correspond to the ones in (E). Bars indicate median with interquartile range. Each dot represents an individual sample. Mann-Whitney *U* test with correction for multiple testing with a False Discovery Rate of 1% was performed.

Hierarchical clustering of the CD4^+^ T cells yielded 18 phenotypically distinct immune cell clusters in the samples that are defined by a unique marker expression profile (Figure 3A). We could distinguish a naïve population (CD45RA^+^CCR7^+^ CD27^+^; cluster 6), antigen-experienced central memory (T_CM_) populations (CD45RO^+^CCR7^+^; clusters 2, 11-13, and 16), effector memory (T_EM_) populations (CD45RO^+^CCR7^-^; clusters 1, 3-6, 8-10, 14, 15, 17, and 18), and terminally differentiated effector memory (T_EMRA_) populations (CD45RA^+^ CD45RO^+/-^CCR7^-^; clusters 5 and 14). Differential expression of HLA-DR, CD38, CD25, PD-1, Tigit, CD161, CD127, CD27, and CD28 was present within the memory compartment. We next evaluated the samples quantitatively by analyzing the frequencies of the CD4^+^ T clusters for all fistula samples (Figure 3B). We did not quantitively assess the rectum samples due to the low sample size. This analysis indicated largely similar compositions of the immune compartment in the CD-associated and cryptoglandular fistulas, with the notable exception of an HLA-DR^+^CD38^+^ effector memory population (Figures 3B and C, cluster 10) that was significantly more abundant (*P* = .0236) in CD-associated fistulas, constituting up to 20% of the CD4^+^ T cells compared with 5% of the CD4^+^ T cells in cryptoglandular fistulas. A significant proportion of these HLA-DR^+^CD38^+^ T_EM_ cells expressed CD25, consistent with cellular activation. Moreover, heterogenous/variable expression of CD69, CD161, CD127, CD28, and PD-1 was observed (Figure 3D).

Hierarchical clustering of the CD8^+^ T cells yielded 16 phenotypically distinct immune cell clusters in the fistula tract and rectum samples. These 16 immune cell clusters consisted of a naïve population (CD45RA^+^CCR7^+^CD27^+^; cluster 2), T_CM_ and T_EM_ populations (CD45RO^+^CD45RA^-^; clusters 3-11, and 13-16), and T_EMRA_ populations (CD45RA^+^CCR7^-^CD45RO^+/-^; cluster 1 and 12; Figure 3E). Analysis of cluster frequencies showed one HLA-DR^+^CD38^+^ CD8 T_EM_ cell population (cluster 13) that was significantly (P = .0372) more abundant in CD-associated than in cryptoglandular fistulas (Figure 3F). A related HLA-DR^-^CD38^+^ cluster (cluster 3) was not significantly different (*P* = .2771) but showed a trend towards a higher frequency in the CD-associated fistula compared with the cryptoglandular. Similar to the HLA-DR^+^CD38^+^ CD4 T_EM_ cell population, these CD8^+^ T cell subsets expressed CD25 and CD69, indicative of cellular activation.

Analysis of the ILC/NK and MNP compartment revealed a trend towards higher frequencies in certain ILC/NK cell and MNP subsets in CD-associated compared with cryptoglandular fistulas. Here, CD56^bright^CD16^-^CD11c^+^ NK-like cells (ILC compartment, cluster 11), dendritic cell types (MNP compartment, cluster 10; CD11c^-^CD123^+^HLA-DR^+^ [pDC] and cluster 20; CD11c^+^HLA-DR^+^CCR7^+^), and a Lin^-^HLA-DR^+^CCR7^+^ cell type (MNP compartment: cluster 8) showed an upward trend in CD-associated fistulas (Supplementary Figure 4A and B). Moreover, an integrated correlation analysis revealed strong correlations between the activated CD4^+^ and CD8^+^ T cell subsets and these innate immune cell subsets (Supplementary Figure 4C).

Together, these data demonstrate the presence of both CD4^+^ and CD8^+^ T_EM_ cell subsets, displaying an activated HLA-DR^+^CD38^+^ phenotype that is significantly increased in CD-associated fistulas compared with cryptoglandular fistulas. The presence of these activated T cells is correlated with HLA-DR^+^ myeloid cell subsets and NK cells in CD-associated perianal fistulas.

### Identification of Disease-associated CD4^+^ and CD8^+^ T Cell Phenotypes in Peripheral Blood of Patients With CD

We next sought to study whether the identified CD-associated HLA-DR^+^CD38^+^ EM CD4^+^ (cluster 10) and (HLA-DR^+^) CD38^+^ EM CD8^+^ (clusters 13 and 3) T cell populations in perianal fistulas could be detected in peripheral blood.

**Figure 4.**
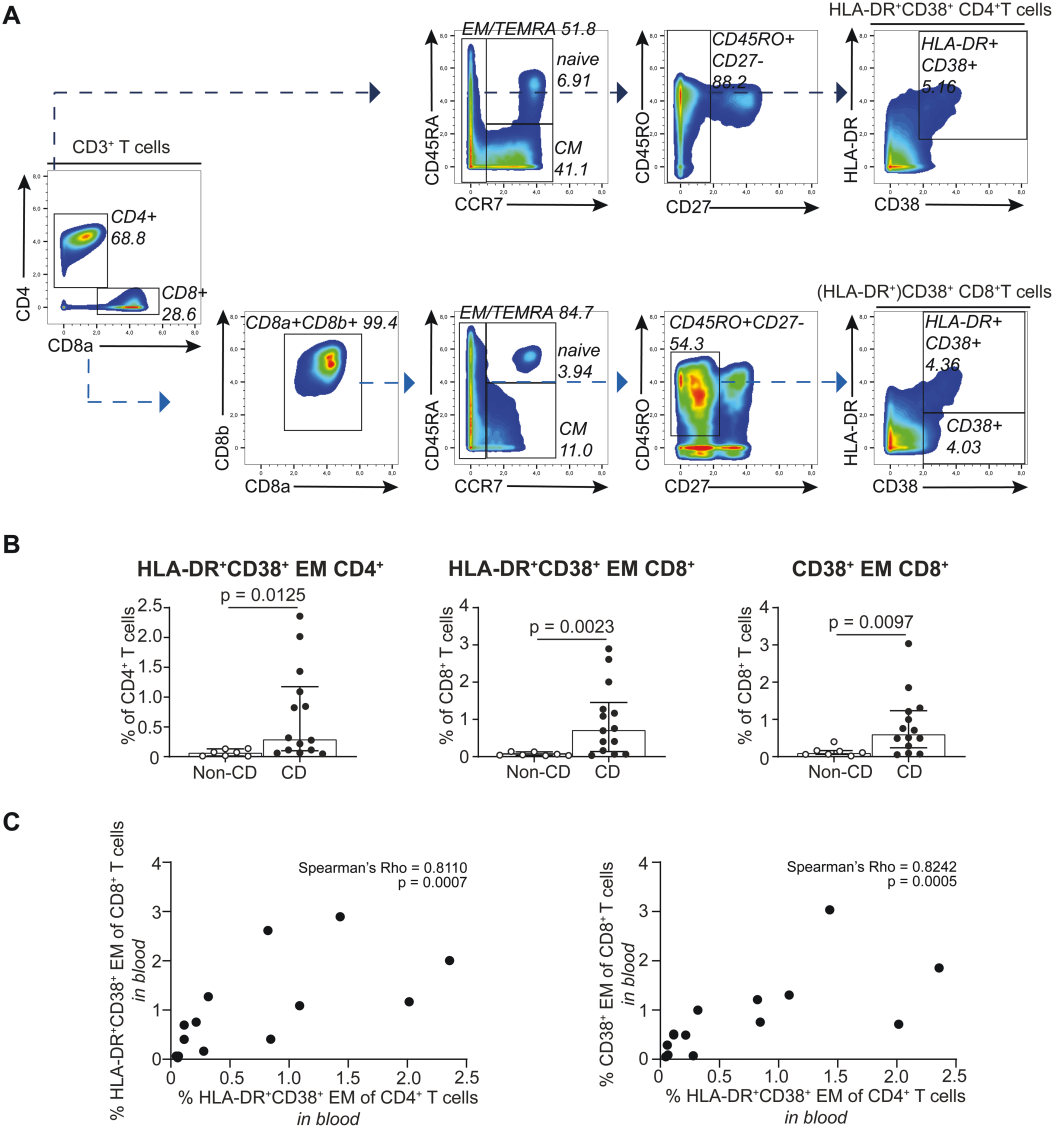
**Identification of disease-associated CD4** ^**+**^  **and CD8**^**+**^  **T cell phenotypes in peripheral blood of patients. A,** Gating strategy used to identify HLA-DR^+^CD38^+^ CD4^+^ and CD8^+^ T cells in blood. B, Frequencies of the HLA-DR^+^CD38^+^ CD4^+^ and CD8^+^ T cells in blood among cryptoglandular fistulas (*n* = 7) and CD-associated fistulas (*n* = 14) as percentage of total CD4^+^ T cells and CD8^+^ T cells, respectively. Bars indicate median with interquartile range. Each dot represents an individual sample. Mann-Whitney *U* test with correction for multiple testing with a False Discovery Rate of 1% was performed. C, Correlation graphs of the HLA-DR^+^CD38^+^ CD4^+^ and CD8^+^ T cells in blood of the 14 CD patients.

Therefore, we analyzed the PBMC samples from non-CD (*n* = 7) and CD (*n* = 14) patients from the first cohort and manually gated on the populations of interest employing the gating strategy as depicted in Figure 4A. Strikingly, this revealed a significantly higher abundance of all 3 (HLA-DR^+^) CD38^+^ cell populations in PBMC from CD patients compared with non-CD patients (Figure 4B). A t-SNE embedding of the HLA-DR^+^CD38^+^ EM CD4^+^ T cell subset in the blood samples revealed a marker expression pattern similar to the equivalent subset in the tract, except for a higher expression of CD69 on the cells in the tract, consistent with tissue residency. Contrarily, CCR6 showed a higher expression on the HLA-DR^+^CD38^+^ EM CD4^+^ T cells in peripheral blood than in the fistula tract (Supplementary Figure 5). Similarly, marker expression overlays of the CD8^+^ T cell populations revealed overlap between the blood and tract counterparts, where the blood counterpart lacked the tissue-residency markers CD103 and CD69. The activation marker CD25 was also more pronounced on both the HLA-DR^+^CD38^+^ CD4^+^ T and CD8^+^ T cell subset in the fistula scrapings compared with their peripheral counterparts (Supplementary Figure 5).

Spearman’s correlation analysis revealed a strong and significant correlation between the CD-associated HLA-DR^+^CD38^+^ EM CD4^+^ T cell phenotype and both the HLA-DR^-^CD38^+^ EM CD8^+^ T cell population (r = 0.8; *P* = .0007) and the HLA-DR^+^CD38^+^ EM CD8^+^ T cell population (r = 0.8; *P* = .0005) in the peripheral samples (Figure 4C).

Taken together, HLA-DR^+^CD38^+^ EM CD4^+^ T cell and HLA-DR^+/-^CD38^+^ EM CD8^+^ T cell populations were identified in blood, where they strongly correlated with each other. These populations discriminated fistula-bearing CD patients from cryptoglandular fistula-bearing patients.

### Th17 CD4^+^ T Cells Are More Activated in Fistulas From CD Patients and Show Skewing Towards a Th1 Phenotype

**Figure 5.**
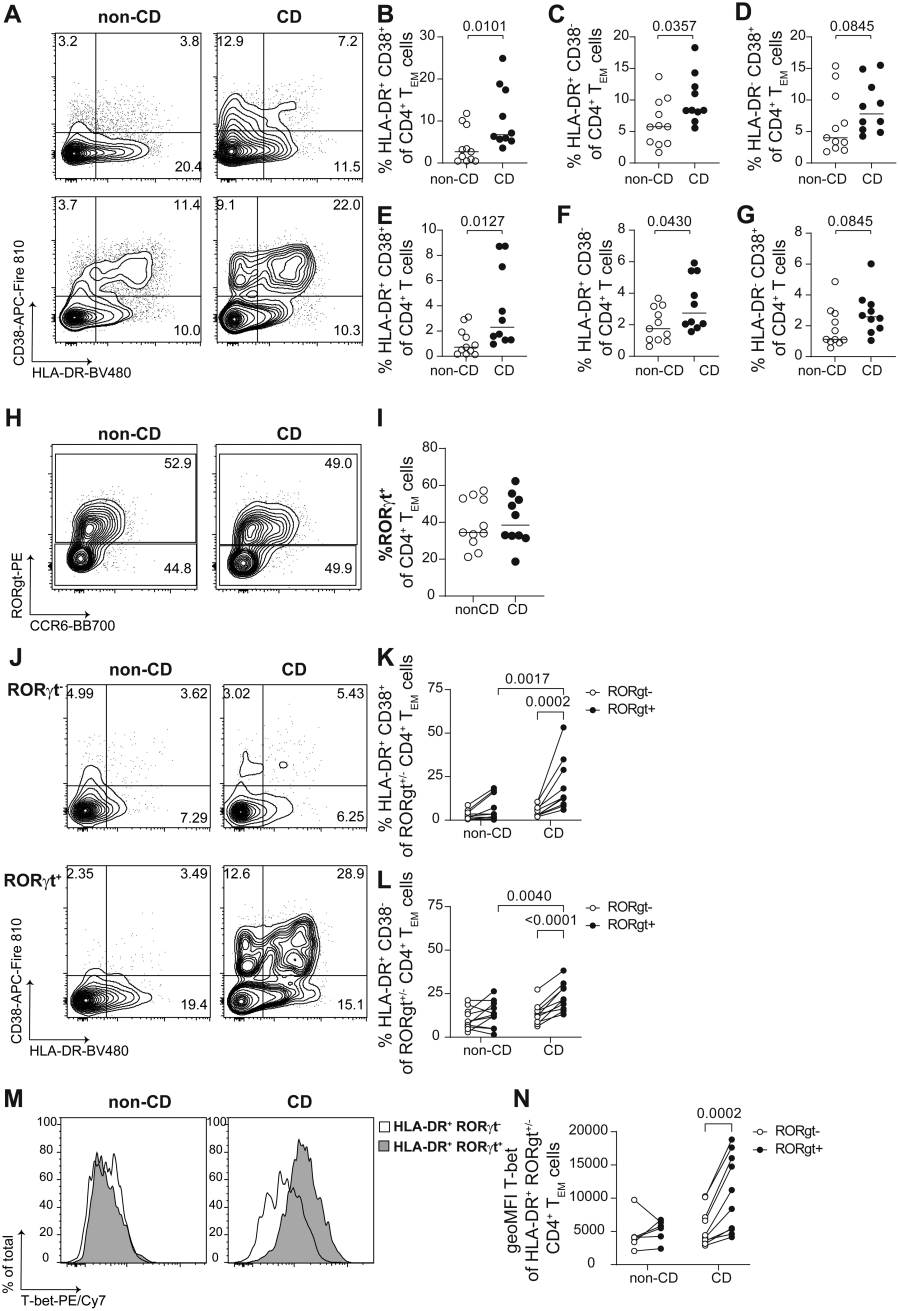
Phenotype of HLA-DR/CD38 subsets from memory CD4^+^ T cell subsets in fistula scrapings. Phenotypic characterization of CD4^+^ T cell subsets in fistula scrapings from patients with cryptoglandular and CD-associated fistulas. (A) Representative plots depicting HLA-DR and CD38 expression in CD4^+^ T_EM_ cells from fistula scrapings from 2 patients with cryptoglandular (left panels) or CD-associated fistulas (right panels). B-D, Frequencies of (B) HLA-DR^+^CD38^+^, (C) HLA-DR^+^CD38^-^ and (D) HLA-DR^-^CD38^+^ cells from CD4^+^ T_EM_ cells; (E-G) Frequencies of (E) HLA-DR^+^CD38^+^, (F) HLA-DR^+^CD38^-^ and (G) HLA-DR^-^CD38^+^ cells from total CD4^+^ T cells. H, Representative plots depicting CCR6 and RORγt expression in CD4^+^ T_EM_ cells from fistula scrapings from a patient with cryptoglandular (left panel) or CD-associated fistula (right panel); (I) frequency of RORγt^+^ cells from CD4^+^ T_EM_ cells; (J) representative plots depicting HLA-DR and CD38 expression in RORγt^-^ (top panels) and RORγt^+^ (bottom panels) CD4^+^ T_EM_ cells from fistula scrapings from a patient with a cryptoglandular (left panel) or CD-associated fistula (right panel); (K,L) Frequencies of (K) HLA-DR^+^CD38^+^and (L) HLA-DR^+^CD38^-^ in RORγt^-^ and RORγt^+^ CD4^+^ T_EM_ cells; (M) histograms showing T-bet expression on HLA-DR^+^ RORγt^-^ (white) and HLA-DR^+^RORγt^+^ (gray) CD4^+^ T_EM_ cells from fistula scrapings from a patient with a cryptoglandular (left panel) or CD-associated fistula (right panel); (N) expression levels of T-bet, shown as geoMFI, on HLA-DR^+^RORγt^-^ (open dots) and HLA-DR^+^RORγt^+^ (black dots) CD4^+^ T_EM_ cells from fistula scrapings from patients with cryptoglandular or CD-associated fistulas. In (K), (L), and (N), values from populations from the same patient are paired and joined by a line. In (B-D) and (E-G), exact *P* values from a Mann-Whitney test are shown. In (K), (L), and (N), exact p values from a paired 2-way ANOVA with multiple comparisons are shown. Cryptoglandular fistula scrapings (*n *= 11, except for panel [N] where samples with <100 HLA-DR^+^ RORgt^+/-^ cells were excluded from the analysis, *n* = 6), CD-associated fistula scrapings (*n* = 10).

Using mass cytometry, HLA-DR^+^CD38 + CD4^+^ T cells emerged as a distinguishing characteristic between CD-associated and cryptoglandular fistulas. We next aimed to determine the helper phenotype of these cells in fistula scrapings from CD and non-CD patients. For this purpose, we developed a 36-antibody SFC panel (Supplementary Table 3) and applied it to single cells prepared from cryopreserved fistula tract scrapings from a new cohort (cohort 2) of CD patients (*n* = 10) and patients without CD diagnosis (*n* = 11). A conventional gating strategy was designed based on the phenotype of the activated cells detected in cohort 1 (Supplementary Figure 6A-B). As observed in the mass cytometry analysis, we detected an HLA-DR^+^CD38^+^CD4^+^ T_EM_ subset in the CD patient samples (Supplementary Figure 6D). Additionally, the increased sensitivity of this technique allowed for the detection of HLA-DR^+^CD38^-^ and HLA-DR^-^CD38^+^ CD4^+^ T_EM_ cells (Figure 5A). Quantification of these subsets in the fistula scrapings revealed that both HLA-DR^+^CD38^+^ and HLA-DR^+^CD38^-^CD4^+^ T_EM_ were increased in frequency in CD-associated fistulas when expressed as percentage of T_EM_ (Figure 5B, C) and as percentage of total CD4^+^ T cells (Figure 5E, F), while HLA-DR^-^CD38^+^ CD4^+^ T_EM_ cells were not significantly different (Figure 5D, G).

In both fistula types, HLA-DR^+^CD38^+^ and HLA-DR^-^CD38^+^CD4^+^ T_EM_ subsets showed higher proportion of Ki-67^+^ cells, which was more pronounced in the former (Supplementary Figure 7A, B). These 2 subsets were also enriched for CD69^+^CD103^-^ and CD39^+^ cells (Supplementary Figure 7C, D), and they displayed lower CD127 and higher CD25 expression when compared with the nonactivated HLA-DR^-^CD38^-^ cells (Supplementary Figure 7E, F). These features were shared to a lesser extent with HLA-DR^+^CD38^-^ CD4^+^ T_EM_ but did not reach statistical significance (Supplementary Figure 7A-F). Regarding their Th phenotype, CD4^+^ T cells in the scrapings of both types of fistula tracts contained a variable proportion of ROR_γ_t^+^ CCR6^lo/-^ cells (Figure 5H, I). The HLA-DR^+^ CD38^+^ and HLA-DR^+^ CD38^-^ cells were significantly increased within the ROR_γ_t^+^ subset in CD-associated fistulas compared with the ROR_γ_t^-^ within the same fistulas and to the ROR_γ_t^+^ cells from the cryptoglandular fistulas (Figure 5J-L). Moreover, T-bet expression was higher in HLA-DR^+^ ROR_γ_t^+^ CD4^+^ T_EM_ cells compared with their HLA-DR^+^ ROR_γ_t^-^ counterparts within CD-associated fistulas (Figure 5M, N).

As observed for the CD4^+^ T cell compartment, we also detected a population of HLA-DR^+^ CD38^+^ within the CD8_αβ_^+^ T_EM_ subset (Supplementary Figure 6C, F, G), as well as HLA-DR^+^CD38^-^ and HLA-DR^-^CD38^+^ cells (Supplementary Figure 8A). These populations were detected at higher levels than those found by mass cytometry (Fig 3F), and no significant differences were observed between the 2 fistula types (Supplementary Figure 8B-G). Of note, HLA-DR^+^CD38^+^ CD8_αβ_^+^ T_EM_ cells showed significantly higher expression of GrzB and Ki-67 than their HLA-DR^-^CD38^-^ counterparts (Supplementary Figure 8H-K).

All in all, these data support the hypothesis that, in both types of fistulas, resident CD4^+^ T_EM_ undergo activation and show features of regulation and differentiation. However, in CD-associated fistulas, activation is mostly detected in CD4^+^ T_EM_ with a mixed Th1/17 phenotype, based on both T-bet and ROR_γ_t expression.

### Heterogeneous Immune Infiltrates Around the Fistula Tract of Crohn’s Disease Patients

**Figure 6.**
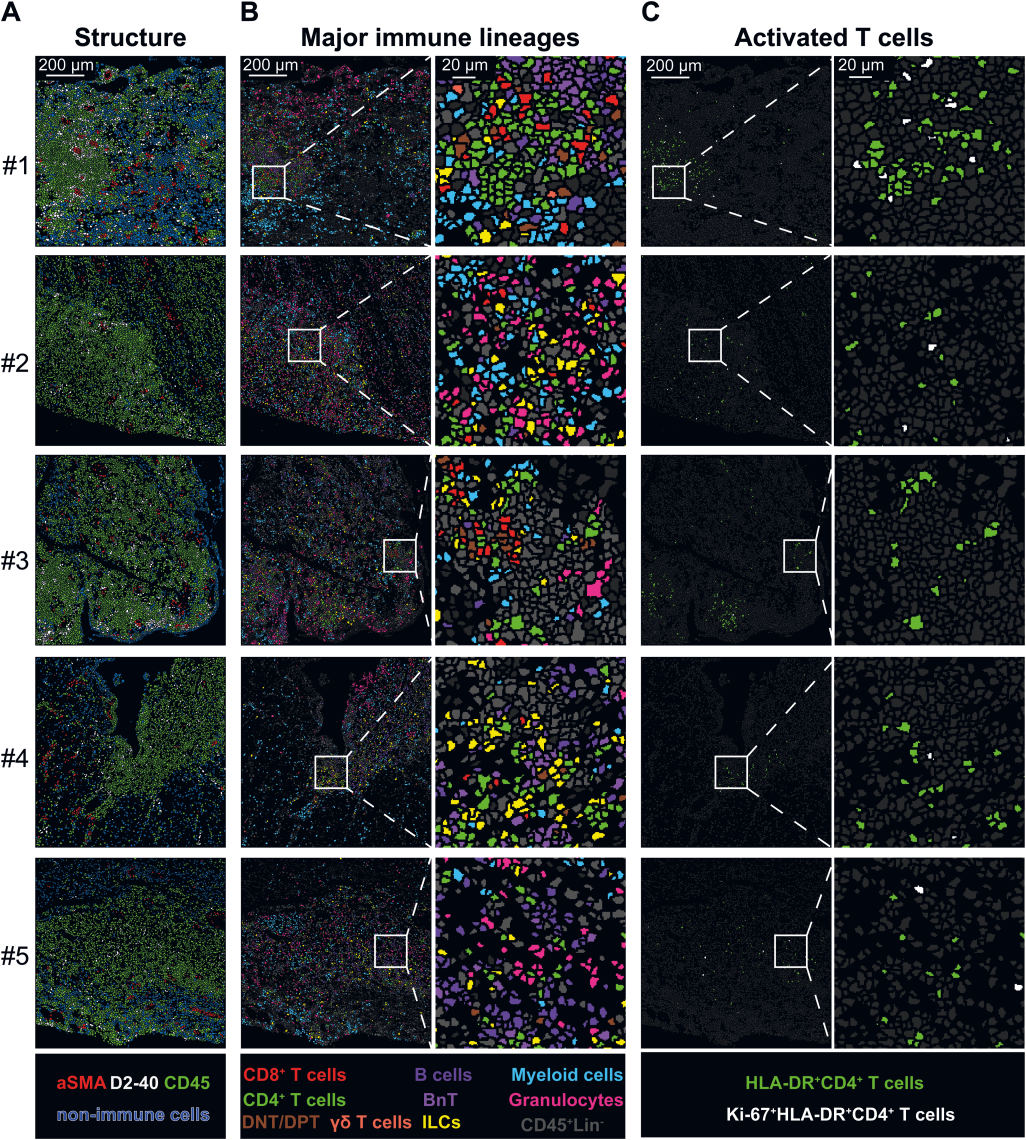
Heterogeneous immune infiltrates around the fistula tract of Crohn’s disease patients. Visualization of the structure (A) and the spatial distribution of the major immune lineages (B) and activated CD4^+^ T cells (C) in one single representative region of interest (ROI) of 5 different patients by imaging mass cytometry. Each color represents a cell type as indicated in the legend. Abbreviations: α-SMA, α-smooth muscle actin; CD, cluster of differentiation; ILC, innate lymphoid cell.

Subsequently, we employed IMC to investigate the cellular neighborhood in which the activated CD4^+^ T cells are residing. For this purpose, we designed a dedicated 33-metal-tagged antibody panel (Supplementary Table 4) and comprehensively characterized fistula tracts from five CD patients (cohort 3). Our selected antibody panel consisted of structural markers (vimentin, alpha-smooth muscle actin [α-SMA], and D2-40), and innate and adaptive immune cell markers (Supplementary Table 4). The IMC data could be quantified similarly to scMC using tSNE by using a cell mask to segment all cells into single cells. Downstream cluster analysis of 32 ROIs in Cytosplore yielded a total of 236 808 cells, of which 152 750 cells were CD45^+^ immune cells. The nonimmune cells comprised mostly of α-SMA^+^ myofibroblasts, D2-40^+^ lymphatic vessels, and nonspecified cells (DNA^+^) lacking CD45 or immune lineage-defining markers, most likely fibroblasts (Supplementary Figure 9A). In Figure 6A, 5 representative ROIs from 5 patients are depicted. Immune cells (in green) were localized close to the tract, whereas nonimmune cells (in dark blue) were localized deeper within the tissue, or lining the tract (Figure 6A).

Within the immune cell compartment, we identified granulocytes, different types of myeloid cells, B cells, T cells, and ILCs (Figure 6B and Supplementary Figure 9B). Within the T/ILC compartment, we distinguished 15 phenotypically distinct immune cell clusters consisting of CD4^+^ T cells, CD8^+^ T cells, double-negative T (CD4^-^ CD8^-^, DNT) cells, TCRγδcells, and ILCs. All CD4^+^ and CD8^+^ T cells expressed CD45RO, and subclusters were formed based on differential expression of HLA-DR, CD57, CCR6, and Ki-67 (Supplementary Figure 9C-E). Importantly, we identified immune cell phenotypes representative of those that were significantly enriched in CD-associated fistulas in our single-cell data set, such as activated CD4^+^ and CD8^+^ T cells (HLA-DR^+^ and Ki-67^+^HLA-DR^+^; Supplementary Figure 9D and E). The HLA-DR^+^ CD4^+^ T cells showed a dispersed or clustered pattern around the tract (Figure 6C) and contained potentially proliferating cells (Ki-67^+^, indicated in white).

As recently described by Hoch et al, we also observed a cluster with cells that simultaneously expressed CD20 and CD3, referred to here as BnT cells.^30^ Myeloid cells (in light blue) were evenly distributed and were prominently present in the immune infiltrates around the tract. With the addition of CD68 and CD163 to this IMC antibody panel, we could identify macrophages. Granulocytes clustered together close to the border of the tract (patients 1, 3, and 4) or were evenly distributed throughout the inflamed tissue (patients 2 and 5).

In summary, IMC allowed for the identification and localization of innate and adaptive immune cells, profiling the immune cell landscape around the tract at great depth. Furthermore, the cell phenotypes observed with imaging mass cytometry closely resembled those found by single-cell mass cytometry. In particular, HLA-DR^+^CD4^+^ T cells could be identified around the fistula tract in a clustered or dispersed manner.

### HLA-DR^+^CD4^+^ T Cells Colocalize With Other CD4^+^ T Cells, B Cells, CD8^+^ T Cells, and Macrophages

Next, we leveraged spatial information to quantify cell-cell interactions between several immune cell types. This enabled us to systematically study the microenvironment of HLA-DR^+^CD4^+^ T cells, including Ki-67^+^ and Ki-67^-^ cells, at the single-cell level. The interactions were determined in the tissue within a 5-pixel (5-um) radius and corrected for differences in cell cluster frequencies using permutation testing,^29^ ruling out that identified interactions do occur at random. An interaction was strong with a z-value of >2.0 (ie, outside 95% normal distribution range) and, on the contrary, was mutually exclusive (ie, the level of interaction is lower than what would be expected based on random chance) with a z-value of <2.0 (Supplementary Table 7).^31^ This allowed us to identify which cells might influence, or are influenced by, HLA-DR^+^CD4^+^ T cells by direct contact and/or secreted products.

**Figure 7.**
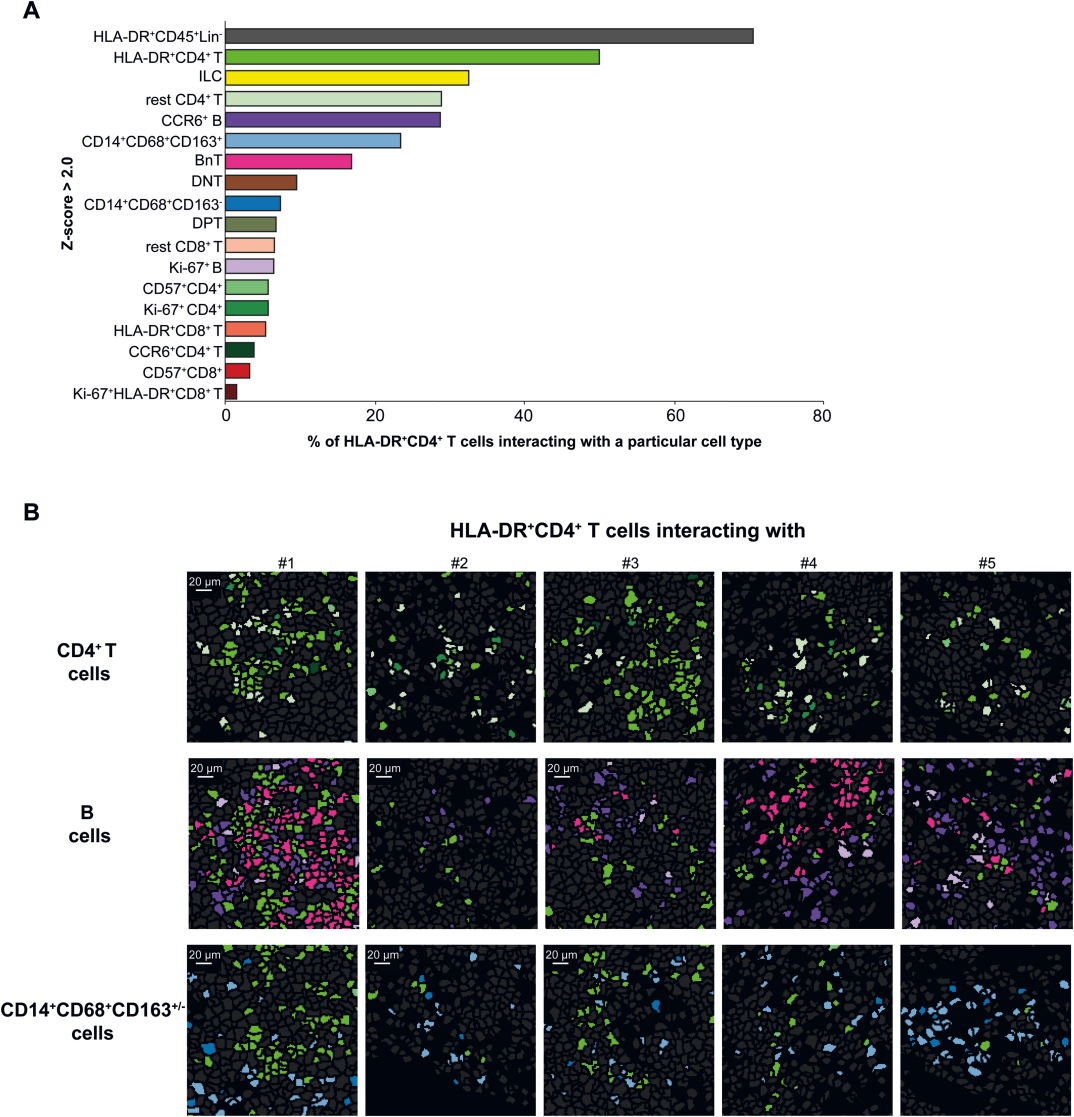
Homotypic and heterotypic cellular interactions of activated CD4^+^ T cells in CD-associated fistula tracts. A, The percentages of HLA-DR^+^CD4^+^ T cells interacting with an immune cell phenotype displayed on the *y*-axis. The immune cell phenotypes that are significantly interacting (Z-score >2.0) with HLA-DR^+^CD4^+^ T cells are shown. B, Visualization of the interaction of HLA-DR^+^CD4^+^ T cells with other CD4^+^ T cells (top row), B cells (middle row), and CD14^+^CD68^+^CD163^+/-^ cells (bottom row). The cell colors match those in panel A.

Eighteen subsets significantly interacted with HLA-DR^+^CD4^+^ T cells (Figure 7A, Supplementary Table 7). Most HLA-DR^+^CD4^+^ T cells resided in the neighborhood of HLA-DR^+^CD45^+^ immune cells, possibly myeloid cells, and cells from the same phenotype. Interestingly, they also colocalized frequently with B cells, most frequently with CCR6^+^ B cells, but also, to a lesser extent, with proliferating (Ki-67^+^) B cells. Moreover, they were also in close vicinity of CD8^+^ T cells, including HLA-DR^+^ CD8^+^ T cells, ILCs, and CD14^+^CD68^+^CD163^+/-^ myeloid cells (Figure 7B). It is noteworthy that HLA-DR^+^CD4^+^ cells were mutually exclusive with granulocytes, other myeloid cells, and CD45^-^ nonimmune cells comprising of vimentin^+^ cells, α-SMA^+^ myofibroblasts, D2-40^+^ lymphatic vessels, and DNA^+^ nonspecified cells lacking CD45 or immune lineage-defining markers (Supplementary Table 7), which indicates that they are located in different cellular environments.

Taken together, spatial cell-cell interaction analysis revealed a close interaction of HLA-DR^+^CD4^+^ T cells with, HLA-DR^+^ CD45^+^ cells, (activated) CD4^+^ T cells, B cells, CD8^+^ T cells, ILCs, and macrophages. Remarkably, considerable proliferation was noted within this neighborhood, including the HLA-DR^+^CD4^+^ T cells, other CD4^+^ T cells, B cells, and CD8^+^ T cells.

## Discussion

Here, for the first time, high-dimensional techniques were applied to comprehensively characterize the innate and adaptive immune cell landscape of CD-associated perianal fistulas and cryptoglandular perianal fistulas at the single-cell level. Single-cell mass cytometry, spectral flow cytometry, and imaging mass cytometry combined allowed us to delineate the cellular diversity in both types of perianal fistulas in-depth. Our analyses revealed a disease-specific signature of activated CD4^+^ T cells with a mixed Th1/17 phenotype and high proliferative capacities in perianal fistulas of patients with CD. We showed that this phenotype colocalized with other CD4^+^ T cells, CCR6^+^ B cells, and macrophages in situ.

Recently, we and others highlighted that HLA-DR^+^CD38^+^ CD4^+^ T cells are associated with intestinal inflammation in a subset of IBD patients.^32,33^ In the present study, we observed the enrichment of HLA-DR^+^CD38^+^ CD4^+^ T cells in virtually all CD-associated fistulas; moreover, in a subset of these patients, this was accompanied by the presence of HLA-DR^+^CD38^+^ CD8^+^ T cells. This may imply that this HLA-DR^+^CD38^+^ T cell phenotype is associated with a more aggressive disease course in CD, resulting in penetrating disease. Activated HLA-DR^+^ CD4^+^ T cells have also been identified in immune-mediated diseases affecting the synovial fluid in juvenile idiopathic arthritis (JIA)^34^ and rheumatoid arthritis (RA) patients.^35^ In JIA patients, activated effector T helper cells are resistant to Treg-mediated suppression, suggesting an important pathogenic role for this activated phenotype. Studying Treg-mediated suppression of HLA-DR^+^CD38^+^ CD4^+^ T cells in the context of IBD would thus be of interest in future studies.

Strikingly, activated HLA-DR^+^CD38^+^ CD4^+^ and CD8^+^ T cells could also be identified in blood and discriminated a subset of fistula-bearing CD patients from cryptoglandular fistula-bearing patients. Of note, in some patients with complex perianal disease the development of fistulas can precede luminal inflammation by several years and sometimes CD is never diagnosed, making it difficult to rationally decide on an appropriate treatment. Therefore, in these patients, monitoring HLA-DR^+^CD38^+^ CD4^+^ and CD8^+^ T cells in blood may have clinical potential to predict CD diagnosis and guide treatment decisions. However, follow-up of the cohort exhibiting cryptoglandular fistulas is essential to corroborate the absence of luminal disease development in these patients. Spectral flow cytometric analysis of fistula samples confirmed the significant increase in HLA-DR^+^CD38^-^ CD4^+^ T_EM_ cells in CD-associated compared with cryptoglandular fistulas. Moreover, this analysis demonstrated higher percentages of HLA-DR^+^ RORγt^+^ CD4^+^ T_EM_ cells in CD-associated fistulas and these HLA-DR^+^ RORγt^+^ cells also displayed higher levels of T-bet, suggesting that activation occurs preferentially in cells with a mixed Th1/17 phenotype. Additionally, Th1/17 cells have been described in the context of actively inflamed gut tissue in patients with CD, where they produce both IL-17A and interferon (IFN)-γ.^36,37^ This further strengthens our hypothesis that activated CD4^+^ T_EM_ cells could help to distinguish CD-associated from cryptoglandular fistulas. The cytokine IL-23 contributes to the polarization of naïve CD4^+^ T cells into IL-17-producing Th17 cells, while IL-12 in turn induces the transformation of the highly plastic Th17 cells into IFN-γ-producing Th1/17 cells.^38^ The distinctive presence of activated Th1/17 cells in CD-associated fistulas suggests that the monoclonal antibody ustekinumab, directly targeting the common p40 subunit of IL-12 and IL-23, could be effective in patients with perianal CD (pCD). In a systematic review, Brewer et al showed perianal improvement after induction with ustekinumab in 110 (31.6%) of 348 patients. Fistula remission was observed in 69 (24.7%) of 279 patients. In the same article, the authors performed a meta-analysis and data from CERTIFI and UNITI trials were included in the post hoc pooled analysis. These randomized controlled data (*n* = 150 patients treated with ustekinumab; *n* = 71 patients treated with placebo) showed at 8-week follow-up a fistula response in 26.0% of patients treated with ustekinumab compared with 16.9% fistula response in placebo patients (*P* = .14). The rate of complete fistula resolution at 8 weeks was 24.7% in ustekinumab-treated patients compared with 14.1% in placebo patients (*P* = .073).^39^ Prospective randomized trials are needed to further elucidate long-term efficacy of ustekinumab and other biologicals for pCD. In the case that effector memory Th1/17 cells do actively produce IFN**-**γ in the fistula tract of CD patients, it remains to be shown whether blocking IL-12/IL-23 can prevent this from happening. Due to the chronic nature of inflammation in the tracts, simultaneously targeting other inflammatory cells, such as fibroblasts and macrophages, together with activated T cells, might help break the cycle of tissue damage.

In the primary mass cytometry data set, we observed that frequencies of both HLA-DR^+^CD38^+^ CD4^+^ T and HLA-DR^+^CD38^+^ CD8^+^ T cells were significantly increased in CD-associated perianal fistulas compared with cryptoglandular fistulas. In a second cohort of patients, spectral flow cytometry was employed, revealing a clear and significant increase in the frequency of HLA-DR^+^CD38^+^ CD4^+^ T cells, distinctly differentiating CD-associated fistulas from cryptoglandular fistulas, and thus confirming the mass cytometry data. However, no significant difference was observed in HLA-DR^+^CD38^+^ CD8^+^ T cells between the 2 patients groups. Nevertheless, although HLA-DR^+^ CD38^+^ CD8^+^ T cells do not distinguish CD-associated from cryptoglandular fistulas, they are present in both types of fistulas and may play a role in the pathogenesis and persistence of fistulas. We observed (Supplementary Figure 8) that this phenotype expresses granzyme B in both CD-associated and cryptoglandular fistulas, suggesting a likely cytotoxic function. It would be of interest to study the functional role of these cells in perianal fistulas in future studies.

To our knowledge, we are the first to investigate the spatial localization of innate and adaptive immune cells in CD-associated fistulas in great depth. Imaging mass cytometry with over 40 immune cell markers simultaneously and coupled to a cell segmentation analysis pipeline^26^ allowed for visualization of complex phenotypes, including the HLA-DR^+^ CD4^+^ T_EM_ cells. Interestingly, HLA-DR^+^ CD4^+^ T_EM_ cells colocalized mainly with cells displaying the same phenotype, other CD4^+^ T cells, and CCR6^+^ B cells. The interaction with B cells has been described in both IBD and other immune-mediated diseases. In seropositive synovial samples from patients with RA, activated PD-1^+^CXCR5^+^ and PD1^+^CXCR5^-^ CD4^+^ T cells, also expressing HLA-DR and CD38, were found adjacent to B cells.^40^ Moreover in CD, Martin et al observed the production of CXCL13, a B cell attractant, in a subset of T cells that expressed HLA-DR and CD38 mRNA in samples from inflamed ileum.^33^ Collectively, these results suggest that activated CD4^+^ T cells could provide B cell help. Furthermore, in one of the fistula tracts, IMC analysis revealed the presence of an organized cluster of cells with a B cell-rich center surrounded by a T cell-rich zone and myeloid cells in the outer layer (Figure 6B #1). This cluster might be an ectopic lymphoid-like structure (ELS), which is linked to deleterious outcomes in certain autoimmune conditions.^41^ In RA, for example, it is associated with inadequate response to anti-TNF.^42^ Concurrently, in inflamed CD lesions, activated CD4^+^ T cells are part of a module that mediates nonresponsiveness to anti-TNF.^33^

Finally, we observed the presence of macrophages in close proximity to the activated HLA-DR^+^ CD4^+^ T cells. Martin et al described chemokine-receptor pairs including CXCL9 and CXCL10 in dendritic cells and macrophages, whose receptor was highly expressed on activated T cells, among others.^33^ Our data show the activated HLA-DR^+^ CD4^+^ T cells also express CXCR3 (Supplementary Figure 7I), suggesting a role for CXCL9/CXCL10 and CXCR3 interaction for attracting macrophages and activated T cells in perianal fistulas in CD patients. Additionally, our observations indicate that proliferating Ki-67^+^ CD4^+^ T cells, CD8^+^ T cells, and B cells colocalize with macrophages and HLA-DR^+^ CD45^+^Lin^-^ cells. These findings suggest that these cell types are all involved in the local activation, expansion, and spatial organization of adaptive immune responses.

In this study, we provide novel insights into the immune cell landscape present in both CD-associated and cryptoglandular fistulas. These findings provide a rationale for future functional studies, which are essential to enhance the understanding of the roles played by these cells and, thereby, to develop potential therapeutic targets. Subsequent studies should prioritize investigating this. The small sample size in this study is a clear limitation. The substantial heterogeneity within the cohort and the small sample size preclude our ability to examine, among other things, the influence of various therapies on immune cell populations and the correlation between the frequency of these activated Th cells with therapy refractoriness and the necessity for a stoma. Hence, in a subsequent study, it would be necessary to collect a larger longitudinal cohort to determine whether these activated Th cells are linked to fistula development in patients with CD.

For the included patients in the cryptoglandular fistula group, a FCP test and endoscopy were not routinely performed during sample collection to rule out CD. However, it is noteworthy that all included patients with a cryptoglandular fistula exhibited a favorable disease course without abnormalities or EIM. In the future, we would recommend both FCP assessment and endoscopy when dealing with cases of recurrent fistulas where CD has not yet been diagnosed.^15^

Crohn’s disease–associated and cryptoglandular perianal fistulas differ substantially in treatment and prognosis. It is therefore essential to find clues to differentiate between them and to develop new targeted therapies for CD patients with treatment-refractory fistulas. In this work, we revealed HLA-DR^+^CD38^+^ CD4^+^ T cells to be exclusively associated with inflammation in CD fistula tracts. The same phenotype was increased in peripheral blood of fistula-bearing CD patients compared with fistula-bearing patients without CD. These HLA-DR^+^CD38^+^CD4^+^ T cells are in close vicinity to B cells and potentially provide B cell help. Targeting HLA-DR and CD38-expressing CD4^+^ T cells may offer a potential new therapeutic strategy for CD-related fistulas.

## Supplementary Data

Supplementary data is available at *Inflammatory Bowel Diseases* online.

izae103_suppl_Supplementary_Tables_1-7_Figures_2-9

izae103_suppl_Supplementary_Tables_3

## Data Availability

The data will be made publicly available upon paper acceptance.

## References

[1] Clayburgh DR, Shen L, Turner JR. A porous defense: the leaky epithelial barrier in intestinal disease. Lab Invest. 2004;84(3):282-291. doi:10.1038/labinvest.3700050 14767487

[2] Friedrich M, Pohin M, Powrie F. Cytokine networks in the pathophysiology of inflammatory bowel disease. Immunity. 2019;50(4):992-1006. doi:10.1016/j.immuni.2019.03.017 30995511

[3] Schwartz DA, Loftus E, Tremaine WJ, et al. The natural history of fistulizing Crohn’s disease in Olmsted County, Minnesota. Gastroenterology. 2002;122(4):875-880. doi:10.1053/gast.2002.32362 11910338

[4] Hellers G, Bergstrand O, Ewerth S, Holmström B. Occurrence and outcome after primary treatment of anal fistulae in Crohn’s disease. Gut. 1980;21(6):525-527. doi:10.1136/gut.21.6.525 7429313 PMC1419665

[5] Schwartz DA, Tagarro I, Carmen Díez M, Sandborn WJ. Prevalence of fistulizing Crohn’s disease in the United States: estimate from a systematic literature review attempt and population-based database analysis. Inflamm Bowel Dis. 2019;25(11):1773-1779. doi:10.1093/ibd/izz056 31216573 PMC6799946

[6] Parks AG. Pathogenesis and treatment of fistula-in-ano. Br Med J. 1961;1(5224):463-469. doi:10.1136/bmj.1.5224.463 13732880 PMC1953161

[7] Whiteford MH. Perianal abscess/fistula disease. Clin Colon Rectal Surg. 2007;20(2):102-109. doi:10.1055/s-2007-977488 20011384 PMC2780182

[8] Malik AI, Nelson RL, Tou S. Incision and drainage of perianal abscess with or without treatment of anal fistula. Cochrane Database Syst Rev. 2010;(7):CD006827. doi:10.1002/14651858.CD006827.pub2 20614450 PMC13270907

[9] Chin Koon Siw K, Engel J, Visva S, et al. Strategies to distinguish perianal fistulas related to Crohn’s disease from cryptoglandular disease: systematic review with meta-analysis. Inflamm Bowel Dis. 2021;28(9):1363-1374. doi:10.1093/ibd/izab286 34792583

[10] Göttgens KWA, Jeuring SFG, Sturkenboom R, et al. Time trends in the epidemiology and outcome of perianal fistulizing Crohn’s disease in a population-based cohort. Eur J Gastroenterol Hepatol. 2017;29(5):595-601. doi:10.1097/MEG.0000000000000840 28350751

[11] Molendijk I, Nuij VJAA, Meulen-De Jong AE van der, et al. Disappointing durable remission rates in complex Crohn’s disease fistula. Inflamm Bowel Dis. 2014;20(11):2022-2028. doi:10.1097/MIB.0000000000000148 25159455

[12] Williams JL, Shaffer VO. Modern management of perianal Crohn’s disease: a review. Am Surg. 2021;87(9):1361-1367. doi:10.1177/0003134820956331 33345571

[13] Sands BE, Anderson FH, Bernstein CN, et al. Infliximab Maintenance Therapy for Fistulizing Crohn’s Disease. *N Engl J Med.* 2004;350(9):876-885. doi:10.1056/NEJMoa030815 14985485

[14] Colombel JF, Sandborn WJ, Rutgeerts P, et al. Adalimumab for maintenance of clinical response and remission in patients with Crohn’s disease: the CHARM trial. Gastroenterology. 2007;132(1):52-65. doi:10.1053/j.gastro.2006.11.041 17241859

[15] Zhou Z, Ouboter LF, Peeters KCMJ, et al. Crohn’s disease-associated and cryptoglandular fistulas: differences and similarities. J Clin Med. 2023;12(2):466. doi:10.3390/jcm12020466 36675403 PMC9860571

[16] Ratto C, Litta F, Lucchetti D, et al. Immunopathological characterization of cryptoglandular anal fistula: a pilot study investigating its pathogenesis. Colorectal Dis. 2016;18(12):O436-O444. doi:10.1111/codi.13527 27649390

[17] van Onkelen RS, Gosselink MP, van Meurs M, et al. Pro-inflammatory cytokines in cryptoglandular anal fistulas. Tech Coloproctol. 2016;20(9):619-625. doi:10.1007/s10151-016-1494-7 27402195 PMC5003909

[18] van Unen V, Li N, Molendijk I, et al. Mass cytometry of the human mucosal immune system identifies tissue- and disease-associated immune subsets. Immunity. 2016;44(5):1227-1239. doi:10.1016/j.immuni.2016.04.014 27178470

[19] Parks AG, Gordon PH, Hardcastle JD. A classification of fistula-in-ano. Br J Surg. 2005;63(1):1-12. doi:10.1002/bjs.1800630102 1267867

[20] Ijsselsteijn ME, Breggen R van der, Sarasqueta AF, et al. A 40-marker panel for high dimensional characterization of cancer immune microenvironments by imaging mass cytometry. Front Immunol. 2019;10:2534. doi:10.3389/fimmu.2019.02534 31736961 PMC6830340

[21] Johnson WE, Li C, Rabinovic A. Adjusting batch effects in microarray expression data using empirical Bayes methods. Biostatistics. 2007;8(1):118-127. doi:10.1093/biostatistics/kxj037 16632515

[22] Höllt T, Pezzotti N, van Unen V, et al. Cytosplore: interactive immune cell phenotyping for large single-cell data sets. Comput Graph Forum. 2016;35(3):171-180. doi:10.1111/cgf.12893

[23] Ma K-L, Santucci G, Wijk JJ van. Hierarchical Stochastic Neighbor Embedding. The Eurographics Association and John Wiley & Sons Ltd.; 2016.

[24] van Unen V, Höllt T, Pezzotti N, et al. Visual analysis of mass cytometry data by hierarchical stochastic neighbour embedding reveals rare cell types. Nat Commun. 2017;8(1):1740. doi:10.1038/s41467-017-01689-9 29170529 PMC5700955

[25] Wei T, Simko V. *R package ‘corrplot’: Visualization of a correlation matrix*. 2021 (Version 0.92). https://github.com/taiyun/corrplot (April 20, 2022, date last accessed).

[26] Ijsselsteijn ME, Somarakis A, Lelieveldt BPF, Höllt T, de Miranda NFCC. Semi-automated background removal limits data loss and normalizes imaging mass cytometry data. Cytometry A. 2021;99(12):1187-1197. doi:10.1002/cyto.a.24480 34196108 PMC9542015

[27] Berg S, Kutra D, Kroeger T, et al. Ilastik: interactive machine learning for (bio)image analysis. Nat Methods. 2019;16(12):1226-1232. doi:10.1038/s41592-019-0582-9 31570887

[28] Carpenter AE, Jones TR, Lamprecht MR, et al. CellProfiler: image analysis software for identifying and quantifying cell phenotypes. Genome Biol. 2006;7(10):R100. doi:10.1186/gb-2006-7-10-r100 17076895 PMC1794559

[29] Somarakis A, Unen V van, Koning F, et al. ImaCytE: visual exploration of cellular micro-environments for imaging mass cytometry data. IEEE Trans Vis Comput Graph. 2021;27(1):98-110. 31369380 10.1109/TVCG.2019.2931299

[30] Hoch T, Schulz D, Eling N, et al. Multiplexed imaging mass cytometry of the chemokine milieus in melanoma characterizes features of the response to immunotherapy. Sci Immunol. 2022;7(70):eabk1692. doi:10.1126/sciimmunol.abk1692 35363540

[31] Abdulrahman Z, Santegoets SJ, Sturm G, et al. Tumor-specific T cells support chemokine-driven spatial organization of intratumoral immune microaggregates needed for long survival. J ImmunoTher Cancer. 2022;10(2):e004346. doi:10.1136/jitc-2021-004346 35217577 PMC8883276

[32] Mitsialis V, Wall S, Liu P, et al.; Boston Children’s Hospital Inflammatory Bowel Disease Center. Single-cell analyses of colon and blood reveal distinct immune cell signatures of ulcerative colitis and Crohn’s disease. Gastroenterology. 2020;159(2):591-608.e10. doi:10.1053/j.gastro.2020.04.074 32428507 PMC8166295

[33] Martin JC, Chang C, Boschetti G, et al. Single-cell analysis of Crohn’s disease lesions identifies a pathogenic cellular module associated with resistance to anti-TNF therapy. Cell. 2019;178(6):1493-1508.e20. doi:10.1016/j.cell.2019.08.008 31474370 PMC7060942

[34] Haufe S, Haug M, Schepp C, et al. Impaired suppression of synovial fluid CD4+CD25- T cells from patients with juvenile idiopathic arthritis by CD4+CD25+ Treg cells. Arthritis Rheum. 2011;63(10):3153-3162. doi:10.1002/art.30503 21702013

[35] Fonseka CY, Rao DA, Teslovich NC, et al. Mixed-effects association of single cells identifies an expanded effector CD4+ T cell subset in rheumatoid arthritis. Sci Transl Med. 2018;10(463):eaaq0305. doi:10.1126/scitranslmed.aaq0305 30333237 PMC6448773

[36] Annunziato F, Cosmi L, Santarlasci V, et al. Phenotypic and functional features of human Th17 cells. J Exp Med. 2007;204(8):1849-1861. doi:10.1084/jem.20070663 17635957 PMC2118657

[37] Ramesh R, Kozhaya L, McKevitt K, et al. Pro-inflammatory human Th17 cells selectively express P-glycoprotein and are refractory to glucocorticoids. J Exp Med. 2014;211(1):89-104. doi:10.1084/jem.20130301 24395888 PMC3892977

[38] Wilson NJ, Boniface K, Chan JR, et al. Development, cytokine profile and function of human interleukin 17-producing helper T cells. Nat Immunol. 2007;8(9):950-957. doi:10.1038/ni1497 17676044

[39] Brewer GMG, Salem G, Afzal MA, et al. Ustekinumab is effective for perianal fistulising Crohn’s disease: a real-world experience and systematic review with meta-analysis. BMJ Open Gastroenterol. 2021;8(1):e000702. doi:10.1136/bmjgast-2021-000702 PMC8685938 34920992

[40] Rao DA, Gurish MF, Marshall JL, et al. Pathologically expanded peripheral T helper cell subset drives B cells in rheumatoid arthritis. Nature. 2017;542(7639):110-114. doi:10.1038/nature20810 28150777 PMC5349321

[41] Pitzalis C, Jones GW, Bombardieri M, Jones SA. Ectopic lymphoid-like structures in infection, cancer and autoimmunity. Nat Rev Immunol. 2014;14(7):447-462. doi:10.1038/nri3700 24948366

[42] Cañete JD, Celis R, Moll C, et al. Clinical significance of synovial lymphoid neogenesis and its reversal after anti-tumour necrosis factor α therapy in rheumatoid arthritis. Ann Rheum Dis. 2009;68(5):751-756. doi:10.1136/ard.2008.089284 18495732

