# Supplementary material for: Activated HLA-DR+CD38+ Effector Th1/17 Cells Distinguish Crohn’s Disease-associated Perianal Fistulas from Cryptoglandular Fistulas
Source: Inflamm Bowel Dis. 2024 May 22;30(11):2146–61. doi: 10.1093/ibd/izae103 (PMC11812577; doi:10.1093/ibd/izae103)
Supplement: izae103_suppl_Supplementary_Tables_1-7_Figures_2-9 [file izae103_suppl_supplementary_tables_1-7_figures_2-9.docx]

**Supplementary Tables and Figures**

**Supplementary Table 1.** Overview clinical samples

| **Single-cell mass cytometry** | | **Non-CD** | **CD** |
| --- | --- | --- | --- |
| Number of patients | | 7 | 14 |
| Total fistula curettage samples | | 5 | 10 |
|  | Paired fistula curettage, rectum biopsy and PBMC, n | 2 | 10 |
|  | Paired fistula curettage and PBMC, n | 3 | 0 |
|  | Paired rectum biopsy and PBMC, n | 2 | 2 |
|  | PBMC, n |  | 2 |
| **Spectral flow cytometry** | | **Non-CD** | **CD** |
| Number of patients | | 11 | 10 |
| Total fistula curettage samples | | 11 | 10 |
| **Imaging mass cytometry** | | **Non-CD** | **CD** |
| Number of patients | |  | 5 |
| CD = Crohn’s disease; PBMC = peripheral blood mononuclear cells | | | |

**Supplementary Table 2.** List of antibodies used for single-cell mass cytometry experiments

|  | **Antibody** | **Metal** | **Clone** | **Supplier** | **Cat#** | **Final  dilution** |  |
| --- | --- | --- | --- | --- | --- | --- | --- |
|  |  |  |  |  |  |  |  |
| 1 | **CD8a** | **^146Nd^** | RPA-T8 | FLM | 3146001B | 1/100 |  |
| 2 | **CD11c** | **^162Dy^** | Bu15 | FLM | 3162005B | 1/200 |  |
| 3 | **CD127** | **^165Ho^** | AO19D5 | FLM | 3165008B | 1/200 |  |
| 4 | **CD38** | **^172Yb^** | HIT2 | FLM | 3172007B | 1/200 |  |
| 5 | **CD69** | **^144Nd^** | FN50 | FLM | 3144018B | 1/100 |  |
| 6 | **CD11b** | **^209Bi^** | ICRF44 | FLM | 3209003B | 1/100 |  |
| 7 | **CD45** | **^89Y^** | HI30 | FLM | 3089003B | 1/100 |  |
| 8 | **CCR6** | **^141Pr^** | G034E3 | FLM | 3141003A | 1/100 |  |
| 9 | **C-Kit** | **^143Nd^** | 104D2 | FLM | 3143001B | 1/100 |  |
| 10 | **CD4** | **^145Nd^** | RPA-T4 | FLM | 3145001B | 1/100 |  |
| 11 | **CD16** | **^148Nd^** | 3G8 | FLM | 3148004B | 1/100 |  |
| 12 | **CD25** | **^149Sm^** | 2A3 | FLM | 3149010B | 1/100 |  |
| 13 | **CD123** | **^151Eu^** | 6H6 | FLM | 3151001B | 1/100 |  |
| 14 | **CD7** | **^153Eu^** | CD7-6B7 | FLM | 3153014B | 1/100 |  |
| 15 | **TIGIT** | **^154Sm^** | MBSA4 | FLM | 3154016B | 1/100 |  |
| 16 | **CCR7** | **^159Tb^** | G043H7 | FLM | 3159003A | 1/100 |  |
| 17 | **CD161** | **^164Dy^** | HP-3G10 | FLM | 3164009B | 1/100 |  |
| 18 | **CD27** | **^167Er^** | O323 | FLM | 3167002B | 1/100 |  |
| 19 | **CD45RA** | **^169Tm^** | HI100 | FLM | 3169008B | 1/100 |  |
| 20 | **CD3** | **^170Er^** | UCHT1 | FLM | 3170001B | 1/100 |  |
| 21 | **PD-1** | **^175Lu^** | EH 12.2H7 | FLM | 3175008B | 1/100 |  |
| 22 | **CD56** | **^176Yb^** | NCAM16.2 | FLM | 3176008B | 1/100 |  |
| 23 | **TCRgd** | **^152Sm^** | 11F2 | FLM | 3152008B | 1/50 |  |
| 24 | **CD40** | **^142Nd^** | 5C3 | FLM | 3142010B | 1/100 |  |
| 25 | **PD-L1** | **^156Gd^** | 29E.2A3 | FLM | 3156026B | 1/200 |  |
| 26 | **CD80** | **^161Dy^** | 2D10.4 | FLM | 3161023B | 1/100 |  |
| 27 | **CD15** | **^115In^** | W6D3 | BioL | 323035 | 1/50 |  |
| 28 | **CD5** | **^160Gd^** | UCHT2 | BioL | 300627 | 1/50 |  |
| 29 | **HLA-DR** | **^168Er^** | L243 | BioL | 307651 | 1/300 |  |
| 30 | **IgM** | **^150Nd^** | MHM88 | BioL | 314527 | 1/100 |  |
| 31 | **CD103** | **^155Gd^** | Ber-ACT8 | BioL | 350202 | 1/100 |  |
| 32 | **CD20** | **^163Dy^** | 2H7 | BioL | 302343 | 1/200 |  |
| 33 | **CD28** | **^171Yb^** | CD28.2 | BioL | 302937 | 1/100 |  |
| 34 | **CD45RO** | **^173Yb^** | UCHL1 | BioL | 304239 | 1/100 |  |
| 35 | **CD122** | **^158Gd^** | TU27 | BioL | 339015 | 1/50 |  |
| 36 | **CD8b** | **^166Er^** | SIDI8BEE | Ebio | 15257407 | 1/50 |  |
| 37 | **NKp46** | **^174Yb^** | 9E 2 | BioL | 331902 | 1/40 |  |
| 38 | **Nkp44** | **^147Sm^** | 253415 | R&D | MAB22491 | 1/40 |  |
| 39 | **CD14** | **^Qdot800^** | TüK4 | Invitrogen/ThermoFisher | Q10064 | 1/1000 |  |
| 40 | **CD57** | **^194Pt^** | HCD57 | BioL | 322325 | 1/200 |  |
| 41 | **CD66b** | **^198Pt^** | 6/40C | BioL | 392902 | 1/40 |  |
| FLM = fluidigm; BioL = biolegend. | | | | | | |  |

|  | **Antigen** | **Fluorochrome** | **Clone** | **Supplier** | **Cat#** | **Dilution** |
| --- | --- | --- | --- | --- | --- | --- |
|  | Human TruStain FcX |  |  | Biolegend | 422302 | 1/20 |
|  | True-stain monocyte blocker |  |  | Biolegend | 426102 | 1/20 |
| 1 | Dead cells | LIVE/DEAD Blue |  | Invitrogen | L34961 | 1/1000 |
| 2 | CD45RA | PerCP | HI100 | Biolegend | 304155 | 1/200 |
| 3 | CD45RO | BV570 | UCHL1 | Biolegend | 304225 | 1/200 |
| 4 | CD25 | BV421 | BC96 | Biolegend | 302630 | 1/25 |
| 5 | CD127 | BV711 | A019D5 | Biolegend | 351328 | 1/25 |
| 6 | CCR4 | BUV615 | 1G1 | BD Biosciences | 613000 | 1/25 |
| 7 | CCR6 | BB700 | 11A9 | BD Biosciences | 566477 | 1/25 |
| 8 | CCR7 | Spark NIR 685 | G043H7 | Biolegend | 353258 | 1/25 |
| 9 | CXCR5 | BV750 | J252D4 | Biolegend | 356942 | 1/25 |
| 10 | CXCR3 | APC/Fire 750 | G025H7 | Biolegend | 353754 | 1/25 |
| 11 | CD103 | PE/Fire 700 | Ber-ACT8 | Biolegend | 350240 | 1/100 |
| 12 | CD38 | APC/Fire 810 | HIT2 | Biolegend | 303550 | 1/25 |
| 13 | CD161 | BUV563 | HP-3G10 | BD Biosciences | 749223 | 1/25 |
| 14 | HLA-DR | BV480 | G46-6 | BD Biosciences | 566154 | 1/200 |
| 15 | CD117 | Vio Bright B515 | REA787 | Miltenyi Biotec | 130-111-674 | 1/200 |
| 16 | CD69 | BUV737 | FN50 | BD Biosciences | 612817 | 1/50 |
| 17 | CD39 | BV650 | TU66 | BD Biosciences | 563681 | 1/25 |
| 18 | CD7 | BUV850 | M-T701 | BD Biosciences | 742002 | 1/50 |
| 19 | CD3 | NovaFluor Blue 660-40S | UCHT1 | Invitrogen | H002T03B07 | 1/25 |
| 20 | CD4 | Alexa Fluor 532 | RPA-T4 | Invitrogen | 58-0049-42 | 1/25 |
| 21 | CD8b | BUV496 | 2ST8.5H7 | BD Biosciences | 749837 | 1/200 |
| 22 | CD8 | Spark Blue 550 | SK1 | Biolegend | 344759 | 1/200 |
| 23 | CD56 | Super Bright 436 | TULY56 | Invitrogen | 62-0566-41 | 1/100 |
| 24 | TCRγ/δ | VioBlue | 11F2 | Miltenyi Biotec | 130-113-507 | 1/100 |
| 25 | TCR Vα7.2 | BV785 | 3C10 | Biolegend | 351722 | 1/50 |
| 26 | CD19 | BV510 | HIB19 | Biolegend | 302242 | 1/25 |
| 27 | CD45 | NovaFluor Blue 610-30S | 2D1 | Invitrogen | H005T03B05 | 1/50 |
| 28 | CTLA-4 | BV605 | BNI3 | Biolegend | 369610 | 1/25 |
| 29 | HELIOS | PerCP-eFluor710 | 22F6 | Invitrogen | 46-9883-42 | 1/25 |
| 30 | FOXP3 | APC | PCH101 | Invitrogen | 17-4776-42 | 1/25 |
| 31 | Ki-67 | BUV395 | B56 | BD Biosciences | 564071 | 1/25 |
| 32 | T-bet | Pe-Cy7 | eBio4B10 | Invitrogen | 25-5825-80 | 1/200 |
| 33 | Gata-3 | PE-Cy5 | TWAJ | Invitrogen | 15-9966-42 | 1/400 |
| 34 | RORγt | PE | Q21-559 | BD Biosciences | 563081 | 1/25 |
| 35 | PU.1 | Alexa Fluor 647 | 7C6B05 | Biolegend | 658004 | 1/25 |
| 36 | BCL-6 | R718 | K112-91 | BD Biosciences | 566979 | 1/50 |
| 37 | Granzyme B | PE/Dazzle 594 | QA16A02 | Biolegend | 372216 | 1/200 |
| PBMCs, UltraComp eBeads (Invitrogen), antii-REA and blank MACS Comp Beads (Miltenyi Biotec were used to record reference controls. | | | | | | |

**Supplementary Table 3.** List of antibodies used for spectral flow cytometry

|  |  |  |  |  |  | **Incubation** | |  |
| --- | --- | --- | --- | --- | --- | --- | --- | --- |
|  | **Antigen** | **Clone** | **Metal** | **Supplier** | **Cat#** | **Time** | **Temperature** | **Dilution** |
| **1** | CD4 | EPR6855 | ^145^Nd | Abcam | ab181724 | Overnight | 4C | 1/200 |
| **2** | TCRgd | H-41 | ^148^Nd | Santa cruz | sc-100289 | Overnight | 4C | 1/50 |
| **3** | CD8a | D8A8Y | ^146^ Nd | CST | 85336BF | 5h | RT | 1/50 |
| **4** | PD-1 | D4W2J | ^160^Gd | CST | 86163BF | 5h | RT | 1/50 |
| **5** | CD103 | EPR4166(2) | ^168^ Er | Abcam | ab221210 | 5h | RT | 1/50 |
| **6** | CD163 | D6U1J | ^173^ Yb | CST | 93498BF | 5h | RT | 1/50 |
| **7** | CD45 | D9M8I | ^175^ Lu | CST | 13917BF | 5h | RT | 1/50 |
| **8** | HLA-DR | TAL 1B5 | ^141^ Pr | Abcam | ab176408 | 5h | RT | 1/100 |
| **9** | CD11b | D6X1N | ^144^ Nd | CST | 49420BF | 5h | RT | 1/100 |
| **10** | Granzyme B | D6E9W | ^150^ Nd | CST | 46890BF | 5h | RT | 1/100 |
| **11** | CD14 | D7A2T | ^163^ Dy | CST | 56082BF | 5h | RT | 1/100 |
| **12** | CD56 | E7X9M | ^167^ Er | CST | 99746BF | 5h | RT | 1/100 |
| **13** | CD7 | EPR4242 | ^174^ Yb | Abcam | ab230834 | 5h | RT | 1/100 |
| **14** | CD11c | EP1347Y | ^176^ Yb | Abcam | ab216655 | 5h | RT | 1/100 |
| **15** | a-SMA | D4K9N | ^209^bi | CST | 19245BF | 5h | RT | 1/100 |
| **16** | PU.1 | 9G7 | ^149^Sm | CST | 2258BF | Overnight | 4C | 1/50 |
| **17** | CD3 | EP449E | ^153^ Eu | Abcam | ab271850 | Overnight | 4C | 1/50 |
| **18** | CD141 | E7Y9P | ^155^ Gd | CST | 43514BF | Overnight | 4C | 1/50 |
| **19** | CD123 | IL3RA/1531 | ^156^ Gd | Abcam | ab268196 | Overnight | 4C | 1/50 |
| **20** | FOXP3 | D608R | ^159^ Tb | CST | 12653BF | Overnight | 4C | 1/50 |
| **21** | CCR6 | ERP22259 | ^162^Dy | Abcam | ab227036 | Overnight | 4C | 1/50 |
| **22** | Vimentin | D21H3 | ^194^ Pt | CST | 5741BF | Overnight | 4C | 1/50 |
| **23** | CD20 | E7B7T | ^142^ Nd | Biolegend | 48750BF | Overnight | 4C | 1/100 |
| **24** | CD68 | D4B9C | ^143^ Nd | cst | 76437BF | Overnight | 4C | 1/100 |
| **25** | CD57 | HNK-1/Leu-7 | ^151^Eu | Abcam | ab212403 | Overnight | 4C | 1/100 |
| **26** | Ki-67 | 8D5 | ^152^ Sm | CST | 9449BF | Overnight | 4C | 1/100 |
| **27** | CD66b | G10F5 | ^154^ Sm | Biolegend | 305102 | Overnight | 4C | 1/100 |
| **28** | CD16 | D1N9L | ^161^ Dy | CST | 24326BF | Overnight | 4C | 1/100 |
| **29** | CD45RO | UCHL1 | ^165^ Ho | CST | 5561BF | Overnight | 4C | 1/100 |
| **30** | D2-40 | D2-40 | ^166^ Er | Biolegend | 916606 | Overnight | 4C | 1/100 |
| **31** | CD38 | EPR4106 | ^169^ Tm | Abcam | ab226034 | Overnight | 4C | 1/100 |
| **32** | CD15 | MC480 | ^171^ Yb | CST | 4744BF | Overnight | 4C | 1/100 |
| **33** | Keratin | C11 | ^198^Pt | Biolegend | 628602 | Overnight | 4C | 1/100 |
| CST: Cell Signaling Technology; RT: room temperature | | | | | | | | |

**Supplementary Table 4.** List of antibodies used for imaging mass cytometry experiments

**
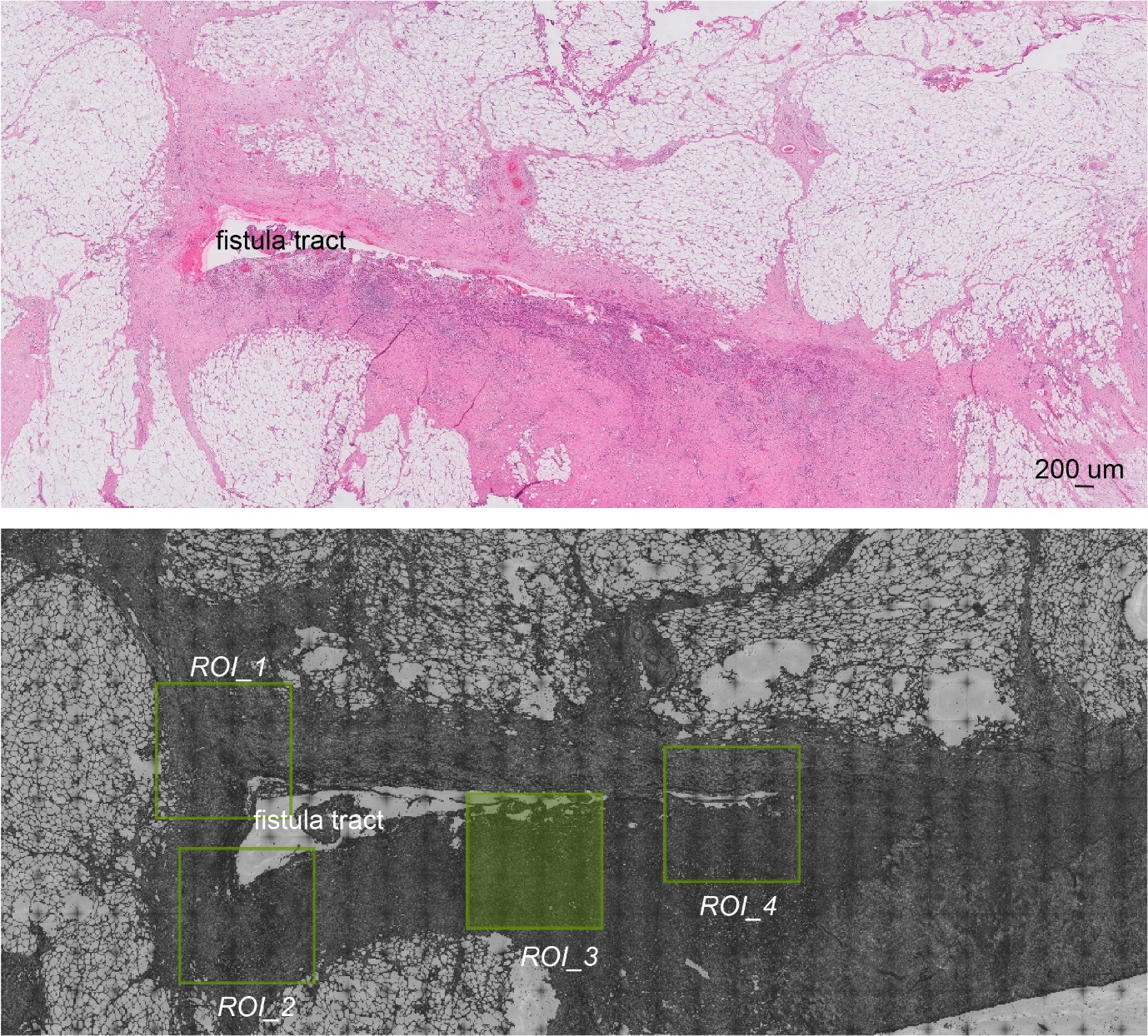

Supplementary Figure 1. Selection of regions of interest along the fistula tract.**Representative H&E stained slide (top) and slide ablated at 200 Hz (bottom) of a fistula tract with the selection of regions of interest (ROI_1, ROI_2, ROI_3, ROI_4).

**Supplementary Table 5.** Patient characteristics, cohort 1

|  | **Non-CD (n=7)** | **CD (n=14)** |
| --- | --- | --- |
| **Age at inclusion in years,** median (IQR) | 52.0 (39.0-58.0) | 41.0 (29.0-45.0) |
| **Sex, female,** n (%) | 3 (42.9) | 12 (85.7) |
| **Smoker,** n (%) |  |  |
| - yes | 1 (14.3) | 2 (14.3) |
| - no | 5 (71.4) | 9 (64.3) |
| - former | 1 (14.3) | 3 (21.4) |
| **Duration of CD in years,** median (IQR) |  | 17.5 (10.5-26.0) |
| **Montreal classification CD,** n |  |  |
| - L1 |  | 2 |
| - L2 |  | 4 |
| - L3 |  | 8 |
| - B2 |  | 11 |
| - B3 |  | 3 |
| **Stoma, n (%)** |  | 5 (35.7%) |
| ***Current use of Biologics****, n |  |  |
| - no | 7 | 2 |
| - anti-TNF (IFX / ADA) | 0 | 4 |
| - anti-IL12/IL23 (UST) | 0 | 4 |
| - anti-α4β7 (VEDO) | 0 | 3 |
| - both anti-α4β7 (VEDO) and anti-IL12/IL23 (UST) | 0 | 1 |
| **Endoscopy for excluding CD diagnosis,** yes | 3 |  |
| **Duration of fistulas in years,** (median (IQR)) | 1.0 (0.0-5.0) | 9.5 (3.5-17.3) |
| **Previous fistula surgery,** n |  |  |
| - 0 | 3 | 3 |
| - 1-2 | 1 | 3 |
| - >5 | 1 | 4 |
| **Route of fistula** with a fistula swab,** n |  |  |
| - Inter-sphincteric | 1 | 2 |
| -Trans-sphincteric | 3 | 2 |
| -Supra-sfincteric |  |  |
| -Extra-sphincteric | 1 | 1 |
| -Inter-sphincteric + trans-sphincteric |  | 1 |
| -Inter-sphincteric + extra-sphincteric |  | 2 |
| -Trans-sphincteric + supra-sphincteric |  | 1 |
| -Trans-sphincteric + extra-sphincteric |  | 1 |
| CD = Crohn's disease; IQR = interquartile range; IFX = infliximab; ADA = adalimumab; UST = ustekinumab; VEDO = vedolizumab.  *all CD patients used steroids, immunomodulator, anti-TNF in the past (11 patients 2 or more classes anti-TNF). 5 patient used ustekinumab in the past and 2 patients vedolizumab. None of the non-CD patients used biologics in the past.  **according to Park’s. | | |

|  | **Non-CD (n=11)** | **CD (n=10)** |
| --- | --- | --- |
| **Age at inclusion in years,** median (IQR) | 44.0 (34.0-54.0) | 41.5 (32.8-53.5) |
| **Sex, female,** n (%) | 3 (27.3) | 4 (40.0) |
| **Smoker,** n (%) |  |  |
| - yes | 2 (18.2) | 2 (20.0) |
| - no | 9 (81.8) | 8 (80.0) |
| - former | NA | NA |
| **Duration of CD in years,** median (IQR) |  | 12.0 (2.8-15.5) |
| **Montreal classification CD,** n |  |  |
| - L1 |  | 3 |
| - L2 |  | 1 |
| - L3 |  | 4 |
| -L3L4 |  | 2 |
| - B2 |  | 9 |
| - B3 |  | 1 |
| **Stoma, n (%)** |  | 0 (0) |
| ***Current use of Biologicals****, n |  |  |
| - no | 11 | 3 |
| - anti-TNF (IFX / ADA) | 0 | 5 |
| - anti-IL12/IL23 (UST) | 0 | 2 |
| - anti-α4β7 (VEDO) | 0 | 0 |
| - both anti-α4β7 (VEDO) and anti-IL12/IL23 (UST) | 0 | 0 |
| **Endoscopy for excluding CD diagnosis,** yes | 1 |  |
| **Duration of fistulas in years,** (median (IQR)) | 1 (1-2) | 6 (4-10) |
| **Previous fistula surgery,** n (%) |  |  |
| - 0 | 6 | 1 |
| - 1-2 | 5 | 0 |
| - 3-5 | 0 | 2 |
| - >5 | 0 | 7 |
| **Route of fistula** with a fistula swab, n** |  |  |
| - Inter-sphincteric | 0 | 1 |
| -Trans-sphincteric | 11 | 4 |
| -Supra-sfincteric | 0 | 2 |
| -Extra-sphincteric | 0 | 1 |
| -Inter-sphincteric + extra-sphincteric | 0 | 1 |
| -Trans-sphincteric + extra-sphincteric | 0 | 1 |
| CD = Crohn's disease; IQR = interquartile range; IFX = infliximab; ADA = adalimumab; UST = ustekinumab; VEDO = vedolizumab.  *9 of 10 CD patients used steroids, immunomodulator in the past. 6 of 10 CD patients used anti-TNF in the past (4 patient 2 or more classes anti-TNF). 1 patient used ustekinumab in the past and 1 patient vedolizumab. 1 patient was just diagnosed and did not use any medication in the past / at inclusion  None of the non-CD patients used biologics in the past. | | |

**Supplementary Table 6.** Patient characteristics, cohort 2

**
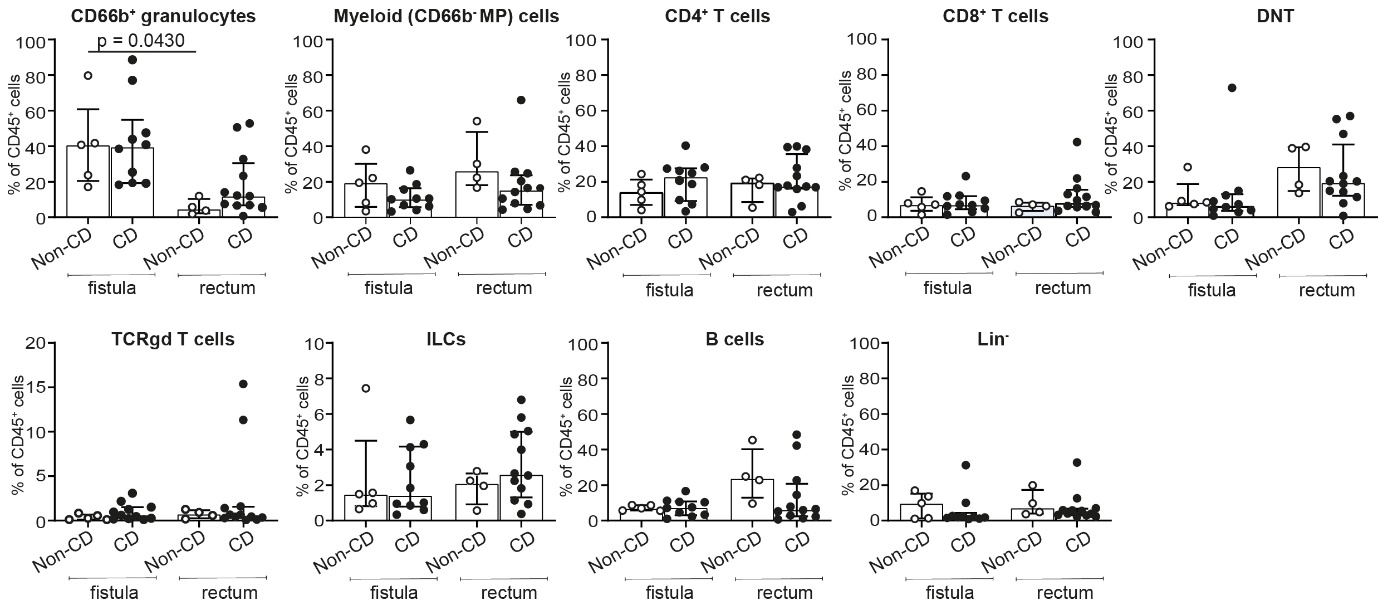
**

**Supplementary Figure 2. Similar composition of the immune compartments in fistula tracts and rectum biopsies from patients with Crohn’s disease and patients without Crohn’s disease.**Frequencies of the major immune lineages in fistula tract samples (CD = 10; non-CD = 5) and rectum biopsies (CD = 12; non-CD = 4). Bars indicate median with interquartile range. Each dot represents an individual sample. Mann-Whitney-U test was performed.

**
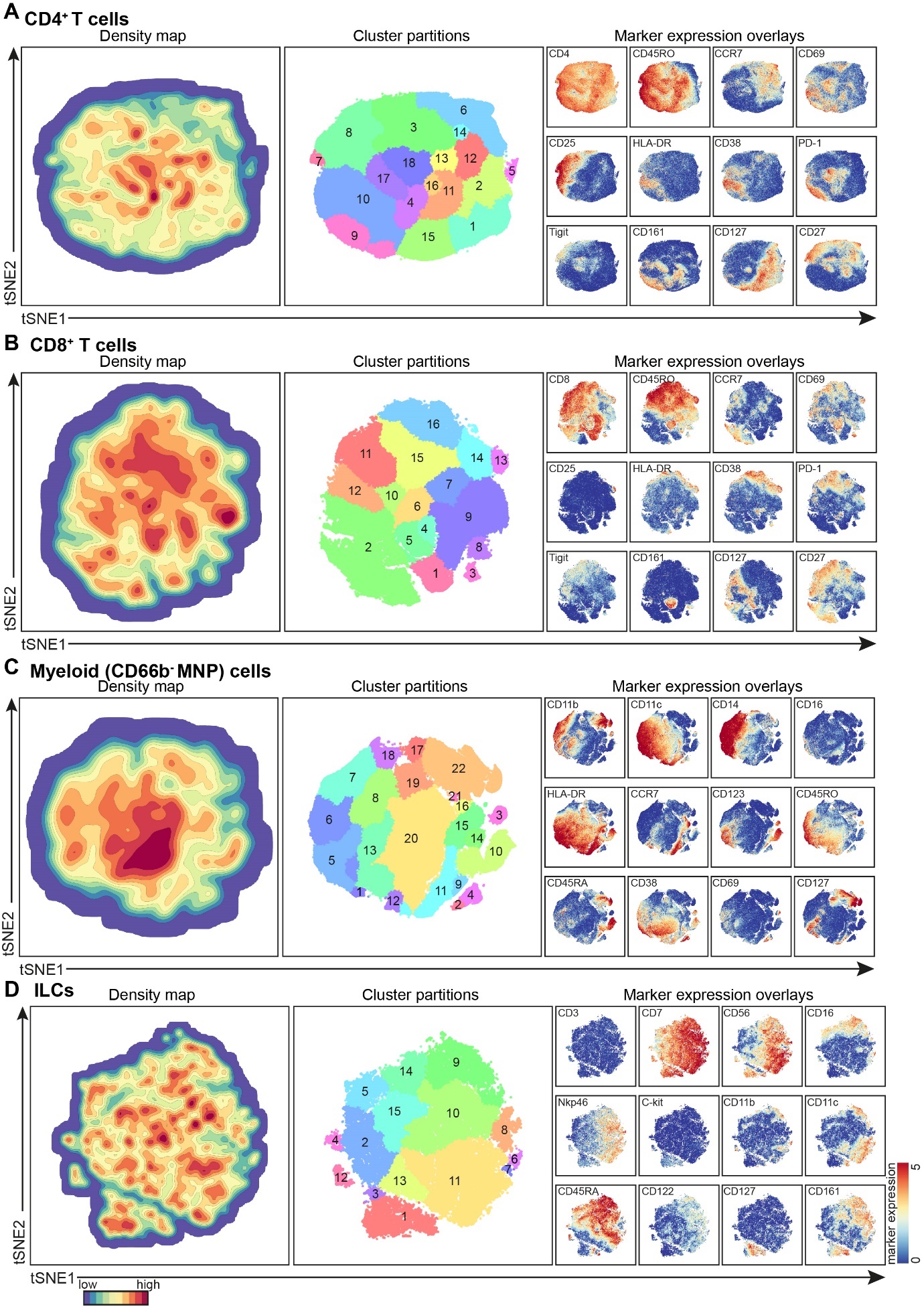

Supplementary Figure 3. t-SNE embedding of CD4^+^ T cell, CD8^+^ T cell, MNP, and ILC compartment**Density maps describing the local probability density of t-SNE-embedded cells using Gaussian mean shift clustering (left), t-SNE plots showing cluster partitions in different colors (middle), t-SNE plots showing relative expression of indicated markers (right) of the **(A)** CD4^+^ T cell compartment (418499 cells), **(B)** CD8^+^ T cell compartment (182608 cells), **(C)** mononuclear phagocyte compartment (309681 cells) and **(D)** innate lymphoid cell compartment (67229 cells). Each dot represents a single cell.

**
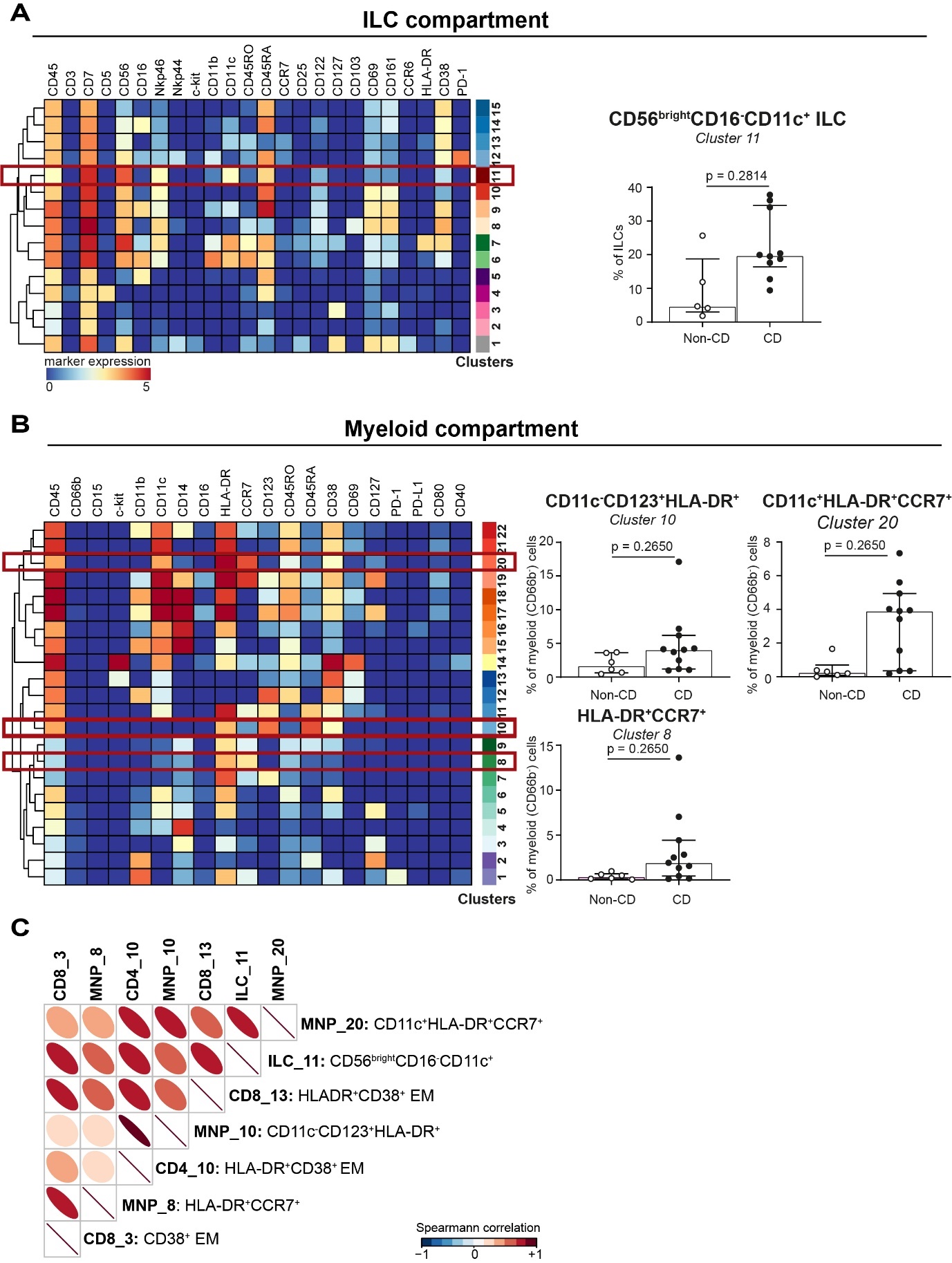

Supplementary Figure 4. Analysis of the ILC/NK and MNP compartment revealed a trend towards higher frequencies in certain ILC/NK cell and MNP subsets in CD-associated compared to cryptoglandular fistulas. (A)** Heatmap (left) of the ILC/NK cell subsets identified through differential expression of the phenotypic markers indicated. The color indicates the median ArcSinh5-transformed marker expression values (blue-to-red scale) of the clusters identified in Fig. S3D (cluster partitions). Frequency (right) of a subset out of a total of 15 ILC/NK cell subsets among cryptoglandular fistulas (n=5) and CD fistulas (n=10) as a percentage of total ILC/NK cells. Cluster number corresponds to the one in the heatmap (left). Bars indicate median with interquartile range. Each dot represents an individual sample. Mann-Whitney-U test with correction for multiple testing with a False Discovery Rate of 1% was performed. **(B)** Heatmap (left) of the MNP subsets identified through differential expression of the phenotypic markers indicated. The color indicates the median ArcSinh5-transformed marker expression values (blue-to-red scale) of the clusters identified in Fig. S3C (cluster partitions). Frequencies (right) of three subsets out of a total of 22 MNP subsets among cryptoglandular fistulas (n=5) and CD fistulas (n=10) as a percentage of total MNPs. Cluster number corresponds to the one in the heatmap (left). Bars indicate median with interquartile range. Each dot represents an individual sample. Mann-Whitney-U test with correction for multiple testing with a False Discovery Rate of 1% was performed. **(C)** Matrix showing correlations (Spearman rank) between seven subsets that are significantly increased or show a trend towards higher frequencies (based on sample frequencies of the corresponding subsets as percentages of total immune cells of that particular lineage) in CD fistulas (n=10) compared to cryptoglandular fistulas (n=5). Color and shape of the ellipses indicate the strength of the correlation.
ILC, innate lymphoid cell; MNP, mononuclear phagocyte.

**
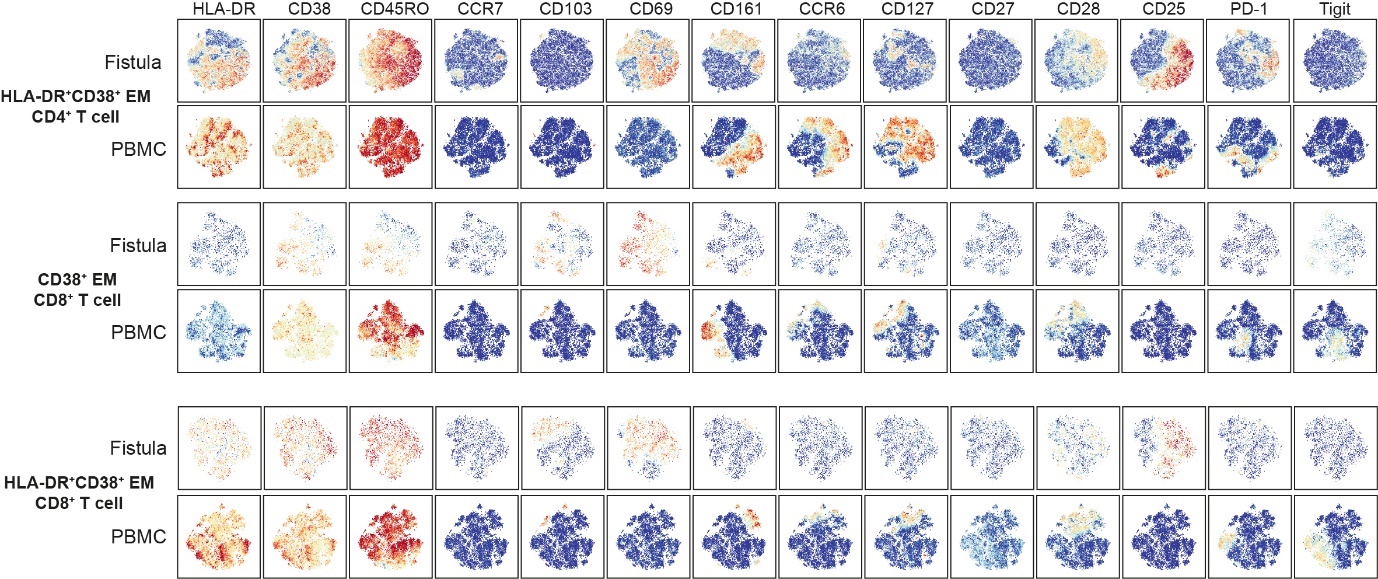
**

**Supplementary Figure 5. Similar marker expression patterns of the disease-associated CD4^+^ and CD8^+^ T cell phenotypes in the fistula tract and blood**t-SNE plots showing relative expression (blue-to-red scale) of indicated markers in fistula samples compared to peripheral blood mononuclear phagocyte samples.
PBMC, peripheral blood mononuclear phagocytes.

**
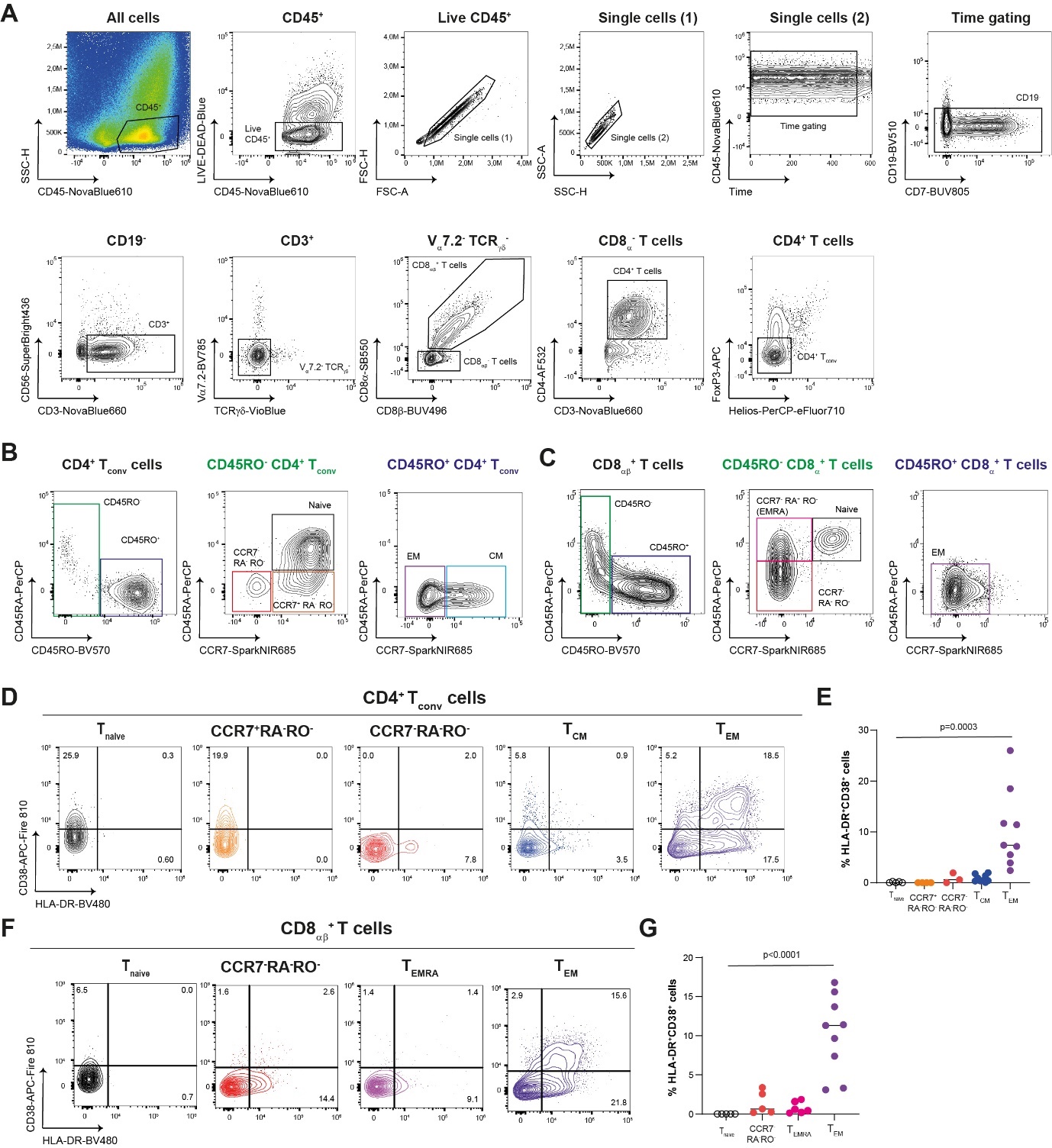
**

**Supplementary Figure 6. Gating strategy for flow cytometry analysis of fistula scrapings**

**(A)** Gating strategy for CD4^+^ and CD8^+^ T cell lymphocytes in fistula scrapings. Lymphocytes were selected first as CD45^+^ cells from a CD45 vs side scatter-area pseudocolor plot. Live cells were subsequently selected from a CD45 vs LIVE/DEAD contour plot. Next, single cells were selected in a forward scatter-area vs forward scatter-height contour plot, followed by a side scatter-height vs side scatter-area contour plot. A contour plot depicting time vs CD45 was later used to gate events within a frame time of stable acquisition, from which T cells were gated sequentially as CD19^-^ CD7^+/^, CD56^-^ CD3^+^, TCR_γδ_^-^ V_α_7.2^-^ cells. From these T cells, conventional CD8^+^ T cells were gated as CD8_α_^+^ CD8_β_^+^ T cells. Conventional CD4+ T cells were gated as CD4^+^ from the CD8_α_^-^ CD8_β_^-^ T cell gate and finally gated as FoxP3^-^ Helios^-^ T cells

**(B)** From the conventional CD4^+^ T cells, CD45RO^+^ and CD45RO^-^ gates were made. CD45RO^-^ conventional CD4^+^ T cells were further divided into CCR7^+^ CD45RA^+^ (Naive), CCR7^+^ CD45RA^-^ (CCR7^+^ RA^-^ RO^-^) and CCR7^-^ CD45RA^-^ (CCR7^-^ RA^-^ RO^-^) cells. CD45RO^+^ cells were divided into CCR7^+^ CD45RA^-^ (central memory, CM) and CCR7^+^ CD45RA^-^ (effector memory, EM) cells.

**(C)** Also, conventional CD8_α_^+^ CD8_β_^+^ (CD8_αβ_^+^) T cells were gated into CD45RO^+^ and CD45RO^-^ cells, which were then further subgated from the CD45RO^-^ cells into CCR7^+^ CD45RA^+^ (Naive), CCR7^-^ CD45RA^-^ (CCR7^-^ RA^-^ RO^-^) and CCR7^-^ CD45RA^+^ (CCR7^-^ RA^+^ RO^-^, EMRA) cells. CD45RO^+^ CD8_α_^+^ CD8_β_^+^ T cells were mostly of an effector memory phenotype (CD45RA^-^ CCR7^-^)

**(D)** Representative plots depicting HLA-DR vs CD38 expression in the different CD4^+^ T cell subsets described in panel B

**(E)** Frequencies of HLA-DR^+^ CD38^+^ within the different CD4^+^ T cells subsets, n=9

**(F)** Representative plots depicting HLA-DR vs CD38 expression in the different CD8_αβ_^+^  T cell subsets described in panel C

**(G)** Frequencies of HLA-DR^+^ CD38^+^ within the different CD8_αβ_^+^ T cells subsets, n=9

**
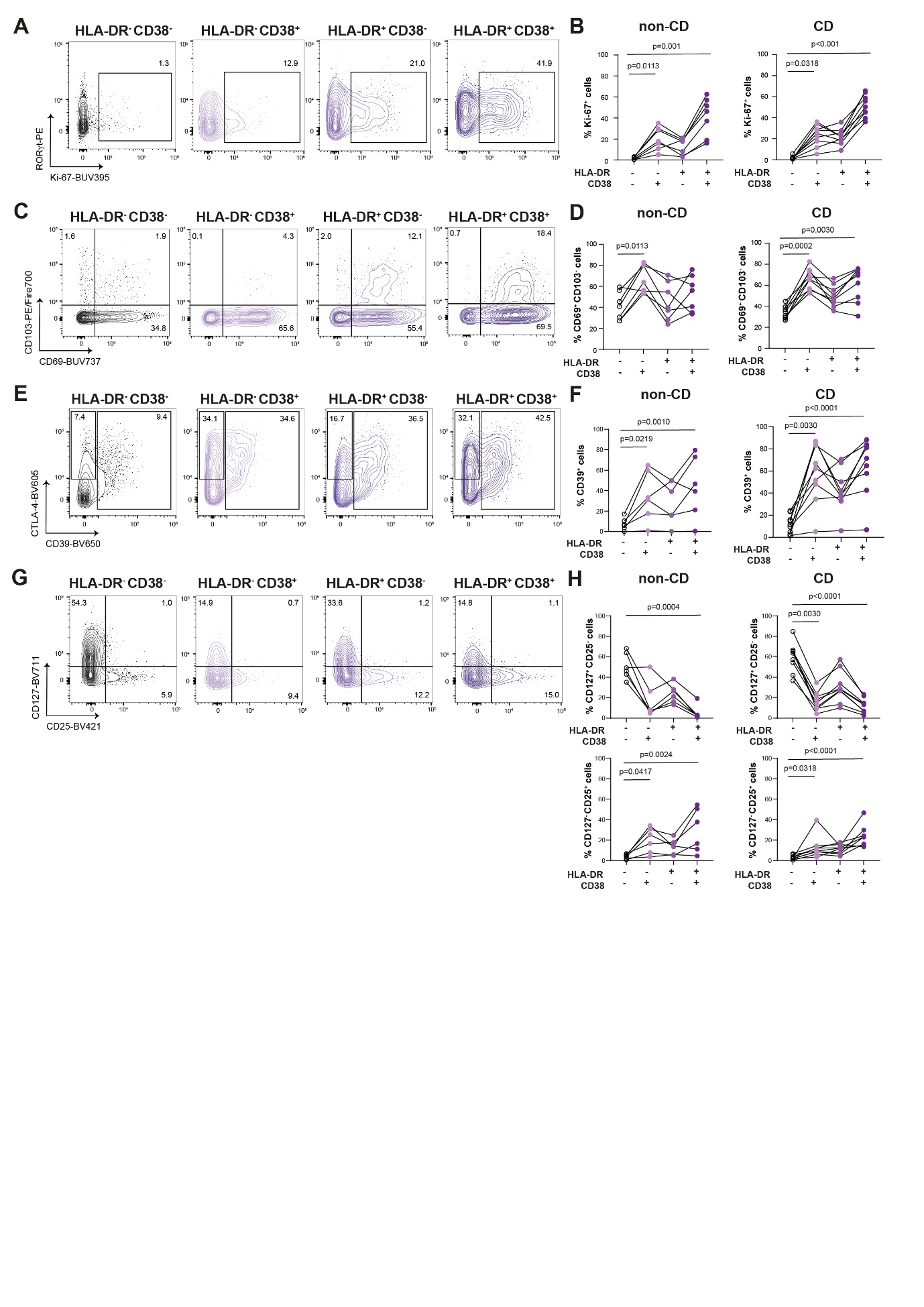
**

**Supplememtal Figure 7. Phenotype of HLA-DR/CD38 subsets from memory CD4+ T cells in fistula scrapings**

**
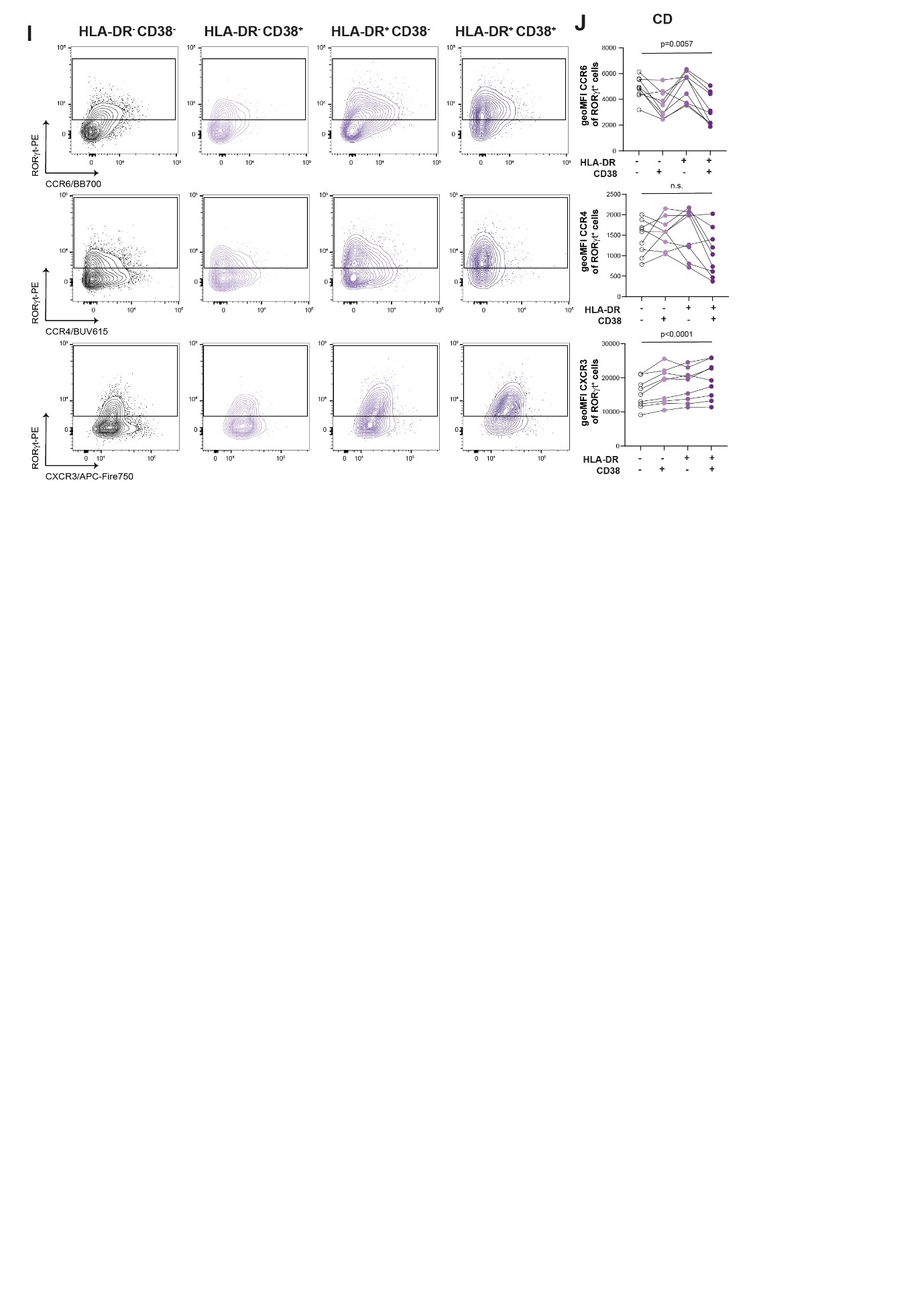
 Supplementary Figure 7 continued. Phenotype of HLA-DR/CD38 subsets from memory CD4+ T cells in fistula scrapings
(A, C, E, G, I)** Representative plots depicting **(A)** Ki-67 vs RORγt, **(C)** CD69 vs CD103, **(E)** CD39 vs CTLA-4, **(G)** CD25 vs CD127 and **(I)** CCR6 vs RORγt (*top*), CCR4 vs RORγt (*center*) or CXCR3 vs RORγt (*bottom*) in HLA-DR^-^ CD38^-^, HLA-DR^-^ CD38^+^, HLA-DR^+^ CD38^-^ and HLA-DR^+^ CD38^-^ subsets of CD4^+^ effector memory T cells in the fistula tracts.

**(B, D, F, H)** Frequencies of **(B)** Ki-67^+^, **(D)** CD69^+^ CD103^-^, **(F)** CD39^+^, **(H)** CD127^+^ CD25^-^ (top) and CD127^-^ CD25^+^ (bottom) cells from the different HLA-DR/CD38 subsets in fistula tracts from non-CD (*left*, n=7) and CD (*right*, n=9) patients.

**(J)** geoMFI of CCR6 (top), CCR4 (center) and CXCR3 (bottom) in cells from the different HLA-DR/CD38 subsets in fistula tracts from CD patients (n=9).

Subsets belonging to the same patient are joined by a line. Values were compared with the Kruskal-Wallis test, followed by Dunn’s multiple comparison. Exact p values are shown for significantly different comparisons.

**
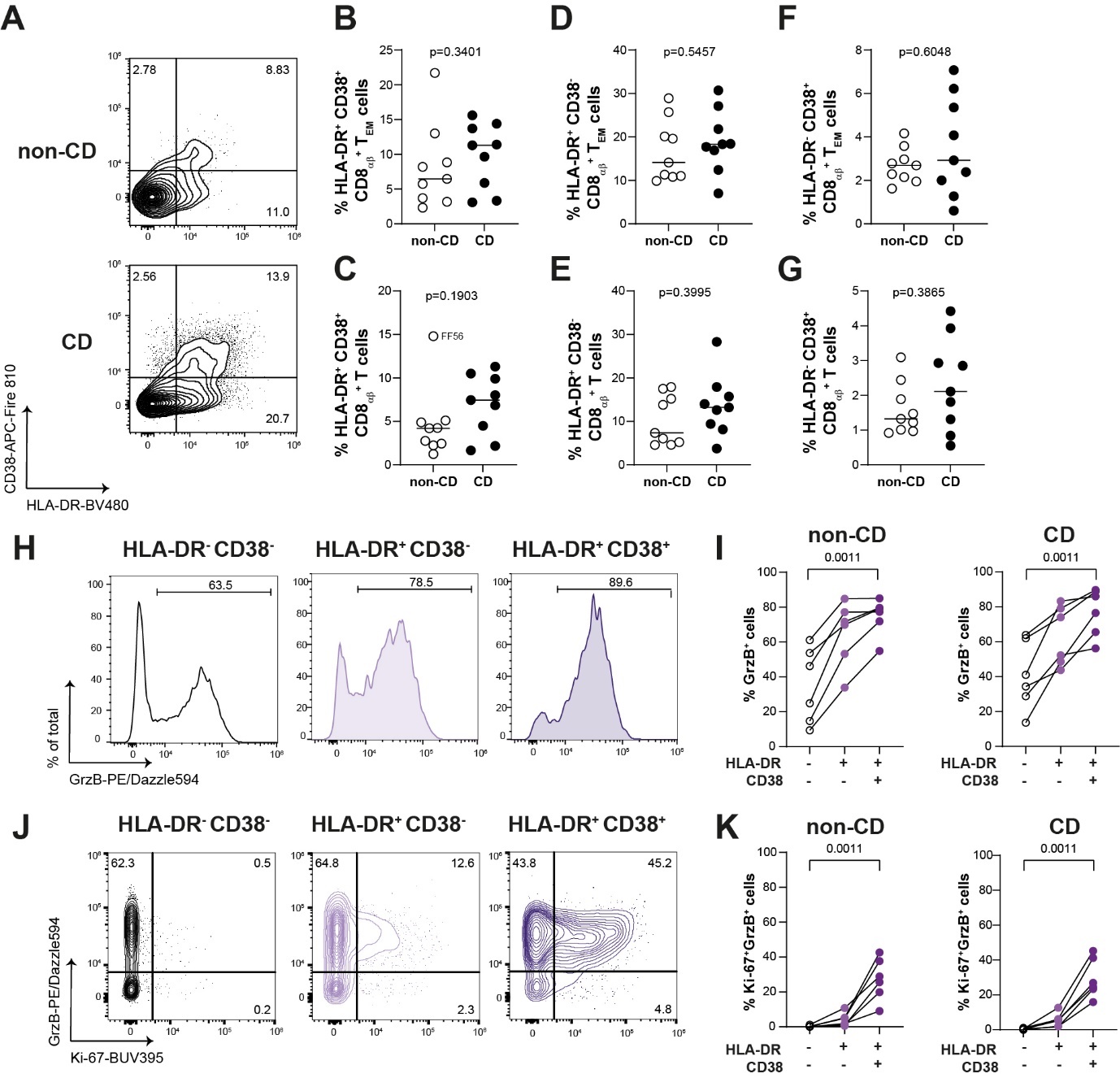

Supplementary Figure 8. Phenotype of HLA-DR/CD38 subsets from memory CD8+ T cells in fistula scrapings**

**(A)** Representative contour plots showing HLA-DR vs CD38 expression in CD8_αβ_^+^ effector memory T cells from the fistula tracts of patients with non-CD (Top) or CD-associated (Bottom) fistulas.

**(B-G)** Frequencies of **(B, C)** HLA-DR^+^ CD38^+^, **(D,E)** HLA-DR^+^ CD38^-^ and **(F,G)** HLA-DR^-^ CD38^+^ cells within (B, D, F) effector memory or (C, E, G) total CD8_αβ_^+^ T cells from fistula scrapings from non-CD (n=8) and CD (n=9) patients. Exact p values from a Mann-Whitney comparison are shown for each comparison.

**(H)** Representative histograms depicting Granzyme B (GrzB) expression on HLA-DR^-^ CD38^-^, HLA-DR^+^ CD38^-^ and HLA-DR^+^ CD38^+^ effector memoty CD8_αβ_^+^ T cells

**(I)** Frequencies of GrzB^+^ cells within the different HLA-DR/CD38 subsets in fistula tracts from non-CD (*left*, n=6) and CD (*right*, n=6) patients.

**(H)** Representative contour plots depicting Ki-67 vs GrzB expression on HLA-DR^-^ CD38^-^, HLA-DR^+^ CD38^-^ and HLA-DR^+^ CD38^+^ effector memoty CD8_αβ_^+^ T cells

**(K)** Frequencies of Ki-67^+^ GrzB^+^ cells within the different HLA-DR/CD38 subsets in fistula tracts from non-CD (*left*, n=6) and CD (*right*, n=6) patients.

For **(I)** and **(K)**, subsets belonging to the same patient are joined by a line. Values were compared with the Kruskal-Wallis test, followed by Dunn’s multiple comparison. Exact p values are shown for significantly different comparisons.

**
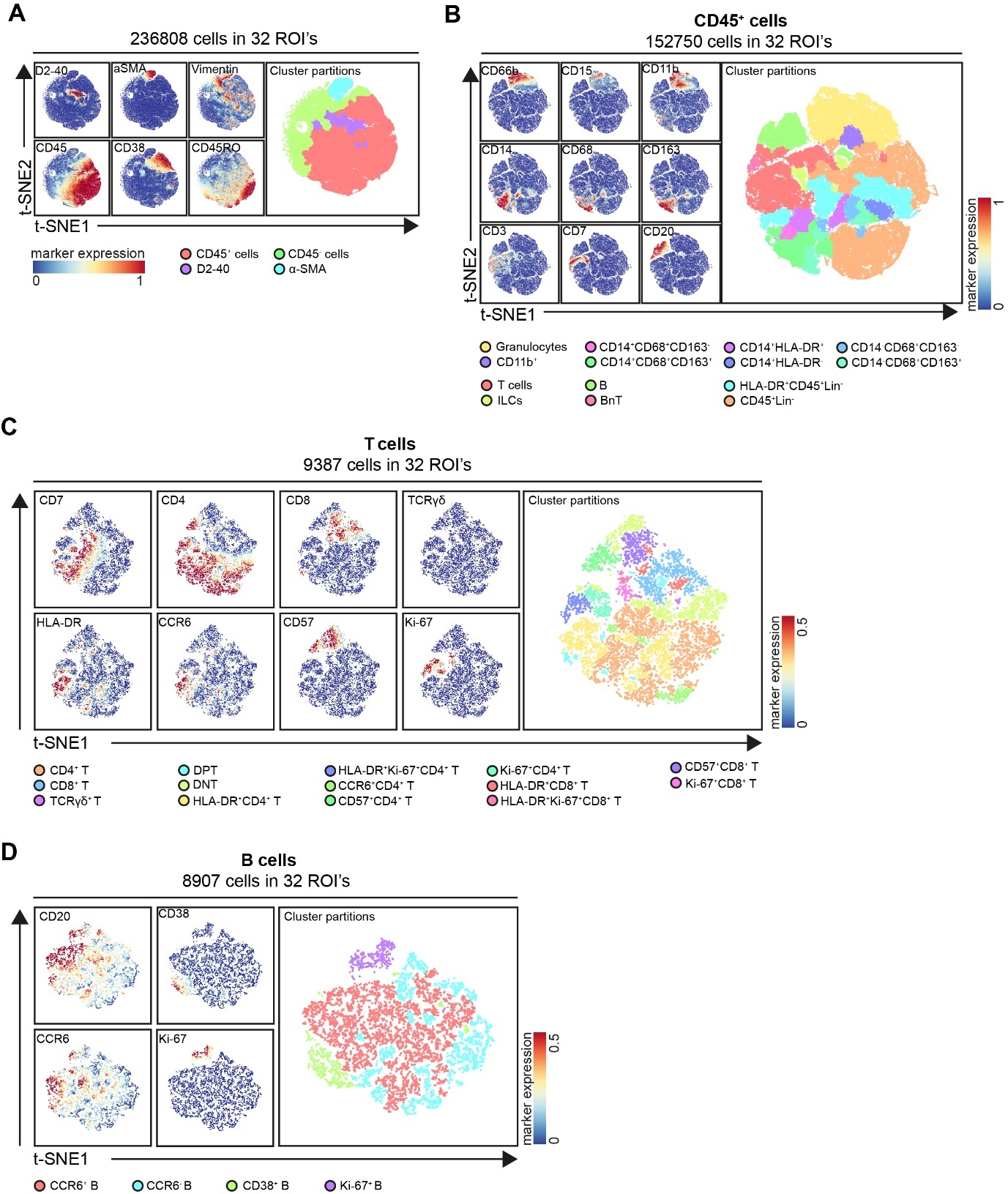

Supplementary Figure 9.** **Identification of innate and adaptive immune cells in fistulas *in situ* using imaging mass cytometry** A collective t-SNE was performed on all DNA^+^ cells **(A)**, then on the selected CD45+ cells **(B)**, the selected T cells **(C)**, and selected B cells **(D)**. Relative marker expression levels (blue-to-red scale) of the indicated markers (left). Colors represent the different levels of marker expression. Cluster partitions in different colors representing different phenotypes as indicated in the legend (right).

| **HLA-DR^+^ CD4^+^ T cell** | | **Cluster interaction** | **Phenotype cluster interaction** | **Z-score** |  |
| --- | --- | --- | --- | --- | --- |
|  |  |  |  |  |  |
| 24 | | **24** | HLA-DR^+^CD4^+^ T | 79.9362 |  |
| 24 | | **12** | HLA-DR^+^CD45^+^Lin^-^ | 43.1728 |  |
| 24 | | **25** | BnT | 42.4221 |  |
| 24 | | **10** | CCR6^+^ B | 21.8144 |  |
| 24 | | **18** | CD4^+^ T | 20.7813 |  |
| 24 | | **23** | DPT | 20.1163 |  |
| 24 | | **21** | ILC | 17.0599 |  |
| 24 | | **26** | CD57^+^CD4^+^ T | 14.1104 |  |
| 24 | | **19** | HLA-DR^+^CD8^+^ T | 13.3975 |  |
| 24 | | **22** | Ki-67^+^CD4^+^ T | 11.2864 |  |
| 24 | | **9** | Ki-67^+^B | 10.8903 |  |
| 24 | | **3** | HLA-DR^-^CCR6^+^CD4^+^ T | 6.79732 |  |
| 24 | | **17** | DNT | 5.26618 |  |
| 24 | | **20** | CD8^+^ T | 4.0579 |  |
| 24 | | **28** | Ki-67^+^HLA-DR^+^CD8^+^ T | 3.80341 |  |
| 24 | | **27** | CD57^+^CD8^+^ T | 3.07793 |  |
| 24 | | **5** | CD14^+^CD68^+^CD163^+^ | 2.40451 |  |
| 24 | | **4** | CD14^+^CD68^+^CD163^-^ | 2.38596 |  |
| 24 | | **32** | Ki-67^+^CD8^+^ T | 0.862194 |  |
| 24 | | **11** | CCR6^-^ B | 0.414581 |  |
| 24 | | **31** | TCRgd | 0.135596 |  |
| 24 | | **8** | CD14^+^HLA-DR^+^ | -1.56161 |  |
| 24 | | **7** | CD14^-^CD68^+^CD163^-^ | -2.91492 |  |
| 24 | | **29** | CD11b^+^ | -4.59147 |  |
| 24 | | **6** | CD14^-^CD68^+^CD163^+^ | -5.01064 |  |
| 24 | | **2** | D2-40 | -5.70499 |  |
| 24 | | **30** | CD66b^+^ granulocytes | -5.71382 |  |
| 24 | | **14** | CD14^+^HLA-DR^-^ | -7.92204 |  |
| 24 | | **1** | aSMA | -13.0697 |  |
| 24 | | **16** | CD14^+^HLA-DR^-^ | -21.1045 |  |
| 24 | | **13** | CD45^-^ | -47.3373 |  |
| Z-score > 2.0 indicates a strong interaction. Z-score < 2.0 indicates that the interaction is mutually exclusive (i.e. the level of interaction is lower than what would be expected based on random chance). | | | | |  |
|  |  |  |  |  |  |

**Supplementary Table 7. Interaction of HLA-DR^+^ CD4^+^ T cells with other phenotypes**
