# Supplementary material for: Activated HLA-DR+CD38+ Effector Th1/17 Cells Distinguish Crohn’s Disease-associated Perianal Fistulas from Cryptoglandular Fistulas
Source: Inflamm Bowel Dis. 2024 May 22;30(11):2146–61. doi: 10.1093/ibd/izae103 (PMC11812577; doi:10.1093/ibd/izae103)
Supplement: izae103_suppl_Supplementary_Tables_3 [file izae103_suppl_supplementary_tables_3.docx]

**Supplementary Methods**

**Sample processing**
Isolation of peripheral blood mononuclear cells (PBMCs) from peripheral blood
Peripheral blood mononuclear cells (PBMCs) were isolated from up to 16 ml of freshly drawn heparin anticoagulated blood. Blood was diluted 2-3 times in phosphate-buffered saline (PBS), layered on 3 mL Ficoll-Paque^TM^ density-gradient, and centrifuged at 2000 rpm for 20 minutes at room temperature (rT) with break zero. The leukocyte layer was collected using a Pasteur pipette, transferred to 20% fetal calf serum (FCS)/RPMI (Gibco, Thermofisher), and washed. Cells were counted, and two million cells were resuspended in staining buffer (Fluidigm) and kept at 4C until antibody staining. The rest of the cells were 1:1 diluted in freezing medium (20%FCS/60%RPMI/20%DMSO) and cryopreserved.

Isolation of leukocytes from rectal biopsies and fistula scraping
*Rectal biopsies*
Intraepithelial lymphocytes (IELs) were isolated from two intestinal biopsies by treatment with 5 ml of HBSS (Sigma-Aldrich) containing 1 mM EDTA (Merck) under rotation for 2 x 45 minutes at 37C. Cells were collected in 1 mL 20% FCS/RMPI and kept at 4C. Next, single cells from the lamina propria (LP) were obtained by enzymatic treatment with a 5 mL ‘enzyme-mix’ containing IMDM culture medium (Lonza) with 20% FCS, 1,000 U/mL collagenase IV (Worthington), and 10 mg/mL DNase I grade II (Roche Diagnostics) under rotation for 1.5 hours at 37C. IELs and LP cells were resuspended, filtered through a 70 µm nylon cell strainer in a 50 mL Greiner tube and mashed with the back of a syringe plunger, and washed with 0.5% FCS/DPBS (Dulbecco’s Phosphate Buffered Saline) (Gibco, Thermofisher) by centrifuging at 1500 rpm for 8 min, brake five. Cells were counted, and a maximum of 2 million cells were resuspended in 1 mL staining buffer (Fluidigm).
*Curettage material*Curettage material of perianal fistulas was collected in 50 ml of HBSS containing 1 mM EDTA and centrifuged at 1500 rpm for 8 minutes with break five. Subsequently, the curettage material was incubated with 15 mL of the ‘enzyme-mix’ as described above. The obtained single-cell suspension was passed with its medium through a 70 µm nylon cell strainer in a 50 mL Greiner tube, mashed with the back of a syringe plunger, and washed with 0.5% FCS/DPBS by centrifuging at 1500 rpm for 8 min, break five. Then washed again by adding 20 mL HBSS + 1mM EDTA by centrifuging at 1500 rpm for 8 min, break five. Subsequently, the cells were incubated with 15 mL of ‘enzyme-mix 2’ containing IMDM culture medium with 20% FCS with 10 mg/mL DNase I grade II by rolling it for 10 minutes at 37C. The cells were passed another time through a 70 µm nylon cell strainer in a 50 mL Greiner tube and washed with a volume of 30 mL 0.5% FCS/DPBS by centrifuging at 1500 rpm for 8 min, break five. Due to a high amount of erythrocytes in fistula scraping, 3 mL of red blood cell lysing solution (BD FACS Lysing Solution 10X (Cat#347691)) were added to the cells and incubated for 2 minutes at room temperature. The cells were washed in 20 mL of 20% FCS/RMPI by centrifuging at 1500 rpm for 8 minutes, break 5. Cells were counted; for CyTOF, a maximum of 2 million cells were resuspended in 1 mL staining buffer (Fluidigm) and freshly stained; for spectral flow cytometry, all cells were 1:1 diluted in freezing medium (20%FCS/60%RPMI/20%DMSO), cryopreserved, and stained at a later timepoint.

**Antibody staining and data acquisition**Single-cell CyTOF
After single-cell isolation, cells were washed with staining buffer (Fluidigm) by centrifuging at 1500 rpm for 8 minutes, break 5, and incubated with 1 mL 2000 mM rhodium DNA intercalator diluted in staining buffer (1:2000) for 15 minutes at room temperature to stain dead cells. Cells were resuspended in 2 mL staining buffer, centrifuged (1500 rpm, 8 min, break 5), and incubated for 10 minutes with Fc Receptor blocking solution (Biolegend). Next, cells were stained with metal-conjugated antibodies (as listed in Table S1) for 45 min at room temperature. After staining, cells were washed three times with staining buffer (1500 rpm, 8 min, break 5) and incubated with 1 mL 500 mM iridium DNA intercalator (Fluidigm) diluted in MaxPar Fix and Perm Buffer (Fluidigm) (1:4000) at 4C overnight, or up to 48 hours, to discriminate single-cells. After incubation, cells were washed three times with staining buffer (2500 rpm, 5 min, brake 5). Before the final wash, cells were counted to adjust cell concentrations for measurement. Before data acquisition, cells were diluted in distilled water containing 1:10 diluted EQ Four Element Calibration Beads (Fluidigm Sciences). Samples were acquired at the Helios mass cytometer (Fluidigm, San Francisco, CA, USA) with the narrow-bore injector at the Flow Core Facility (FCF) of the Leiden University Medical Center. CyTOF data were acquired and analyzed on-the-fly, using dual-count mode and noise-reduction on. All other settings were either default settings or optimized with tuning solution, as instructed by Fluidigm. After data acquisition, the mass bead signal was used to normalize the short-term signal fluctuations with the reference EQ passport P13H2302 during the course of each experiment. Samples from the same patient were processed and acquired the same day.

Spectral flow cytometry
Cryopreserved cells isolated from fistula curettage material were thawed and rested for 10 min at 37℃. Next, cells were resuspended in 10% FCS (Bodinco BV) /IMDM (Gibco) with 200 µg/mL DNase I (Roche) and incubated for 10 min at 37℃ while rolling. Next, cells were passed through a 35 µm nylon mesh strainer (Falcon). Staining for the 37-color flow cytometry panel was divided over 4 steps (Supplementary Table 3). To stain surface markers, cells were incubated with the first antibody mixture in 1% FCS/PBS (Fresenius Kabi) for 30 min at 4℃ and were washed twice with 1% FCS/PBS. This process was subsequently repeated with the 2^nd^ and 3^rd^ antibody mixtures. Cells were fixated and permeabilized using the Foxp3 / Transcription Factor Staining Buffer Set (Invitrogen). After surface staining, cells were incubated in Fixation/Permeabilization solution for 30 min at 4℃ and washed with Permeabilization Buffer. To stain intracellular proteins, cells were incubated with the 4^th^ antibody mixture in Permeabilization Buffer for 45 min at 4℃ and were washed twice with Permeabilization Buffer. Finally, cells were resuspended in 1% FCS/PBS and passed once more through a 35 µm nylon mesh strainer. Samples were acquired the same day on a 5-laser Cytek Aurora spectral flow cytometer (Cytek Biosciences), using SpectroFlo Software, in the FCF of the LUMC. Data was unmixed with previously recorded reference controls and autofluorescence extraction was enabled.

Imaging mass cytometry
A 33-antibody panel (Supplementary Table 3) was designed with antibodies pre-conjugated or conjugated in-house. Antibody specificity prior to and post metal-conjugation as well as optimal antigen retrieval conditions were assessed by immunohistochemistry (IHC) on 4 um tonsil tissue and non-inflamed colon tissue.
The start-up of the Hyperion was performed as previously described^1^. A total of 32 1um^2^ regions of interest (ROIs) along the fistula tract were selected across 8 samples from 5 patients (3 patients: one tissue block; 1 patient: two tissue blocks, same fistula tract, same time point; 1 patient: three tissue blocks, same fistula tract, same time point) based on a consecutive H&E stained slide and ablated at 200 Hz (Supplementary Figure 1; top: H&E stain, bottom: ablation slide). Tissue sections were ablated within a month after immunodetection by the Hyperion mass cytometer imaging system (Fluidigm, San Francisco, CA, USA) at the Flow cytometry Core Facility (LUMC, Leiden, the Netherlands). Data were exported as MCD files and txt files and visualized using Fluidigm MCD TM viewer. For downstream analysis, the MCD files were transformed to either 32-bit multi-tiff or single-marker tiff images in the MCD TM viewer software. An imaging processing pipeline was used to generate cell masks to convert raw IMC images into single-cell data^1^.

**References**

1. Ijsselsteijn ME., Somarakis A., Lelieveldt BPF., Höllt T., de Miranda NFCC. Semi-automated background removal limits data loss and normalizes imaging mass cytometry data. *Cytometry Part A* 2021. Doi: 10.1002/cyto.a.24480.
